# Pollution removal efficiency enhancement by agricultural biomass additions in constructed wetlands: A framework integrating meta-analysis with explainable machine learning

**DOI:** 10.1371/journal.pone.0353064

**Published:** 2026-07-07

**Authors:** Wenjie Li, Jian Wang, Xinya Liu, Yulin Zhuang, Hongda Fang, Jinliang Huang

**Affiliations:** 1 Fujian Key Laboratory of Coastal Pollution Prevention and Control, College of the Environment and Ecology, Xiamen University, Xiamen, China; 2 School of Economics, Xiamen University, Xiamen, China; 3 College of Chemistry, Chemical Engineering and Environmental Science, Minnan Normal University, Zhangzhou, China; 4 College of Habour and coastal Engineering Jimei University, Xiamen, China; Indian Institute of Technology Delhi, INDIA

## Abstract

The agricultural biomass addition on constructed wetlands (CWs) is a sustainable strategy that integrates pollution control with waste valorization. However, their widespread applications remain constrained by variations in biomass types, pretreatments, and system designs. This study combined meta-analysis and explainable machine learning to assess how agricultural biomass addition influences Chemical Oxygen Demand (COD) and Total Nitrogen (TN) removal in CWs, particularly under low C/N conditions, using 272 and 1,283 independent observations for meta-analysis and machine learning, respectively. Results showed that bamboo biochar significantly enhances COD removal efficiency in CWs by an average of 54.8% (SMD = 2.50), while lotus leaf biochar improves TN removal efficiency by 32.5% (SMD = 1.20) compared with controls. Additionally, multiple machine learning models were tested and the results showed that the XGBoost model demonstrated the robustperformance in simulating TN removal (R^2^ = 0.83), whereas the Random Forest model is effective for COD removal (R^2^ = 0.76). SHAP analysis further indicatedthat increasing wetland volume can simultaneously enhance both COD and TN removal efficiencies. This study underscores the power of combining meta-analysis and explainable machine learning to optimize CW design and management, offering a robust framework for improving pollution removal in CWs.

## Introduction

Constructed wetlands (CWs) are recognized as environmentally friendly and cost-effective natural water treatment systems [1,2]. They emulate the structure and function of natural wetlands by channeling wastewater through an integrated ecosystem composed of substrates, vegetation, fauna, and microbial communities [3]. Over the past two decades, CWs have been extensively employed worldwide, not only for municipal wastewater treatment but also for managing industrial and agricultural wastewater, landfill leachate, and stormwater runoff [4].

The resource-oriented utilization of rural waste in CWs represents a sustainable strategy for enhancing denitrification while addressing environmental challenges associated with agricultural residues. This approach supports the principles of the circular economy by transforming waste into value-added resources, thereby promoting ecological balance and reducing dependency on synthetic carbon sources. For example, Awasthi et al. [5] highlighted that the prevailing linear economic model of “take-make-dispose” has led to severe resource depletion and rural waste accumulation, underscoring the urgency of sustainable waste reuse. Among various biomass-based materials, biochar has attracted increasing attention because of its high porosity, large surface area, and cation-exchange capacity, which facilitate pollutant adsorption, microbial colonization, and contaminant degradation in CWs. Beyond wastewater treatment, biochar serves as an effective soil amendment, improving nutrient retention, microbial activity, and water-holding capacity, thus extending its value to broader environmental applications [6].

Agricultural biomass has emerged as a promising alternative to conventional carbon sources in CWs, simultaneously enhancing nitrogen removal efficiency and promoting rural waste valorization [7]. For example, Jia et al. [8] demonstrated that recycled agricultural biomass can serve as an effective and low-cost carbon source, improving nitrogen removal efficiency. Similarly, Li et al. [9] and Zhong et al. [10] reported that integrating rural biomass, such as corn straw or biochar, into CWs not only supports microbial denitrification but also contributes to resource recycling. Ajibade et al. [11] further observed that biochar-amended CWs significantly enhanced TN removal under low C/N conditions. Collectively, these findings highlight the feasibility and environmental benefits of integrating CW technology with rural waste valorization, aligning both ecological and economic goals within the circular economy framework.

Therefore, systematic and data-driven approaches to are crucially required to evaluate the role of agricultural waste as a carbon source for nitrogen removal in CWs. While traditional experimental designs are valuable, they are often limited in scalability, comparability, and reproducibility when applied to diverse waste types and wetland configurations. For example, Tao et al. [12] investigated the use of agricultural residues, such as reeds, as external carbon sources and observed enhanced denitrification efficiency in CWs treating low C/N wastewater. However, these studies are typically constrained by site-specific conditions and lack cross-study generalizability. Integrating existing research through meta-analysis and predictive modeling offers a promising solution for overcoming these challenges.

Meta-analysis involves the statistical integration of findings from individual studies on a unified scale and has been applied in research on CWs, providing a foundation for further research [13]. For instance, Yan et al. [14] used meta-analysis to assess the factors influencing trace organic contaminant removal in CWs and found that removal patterns correlated with CW conditions, with multiple parameters determining removal efficiency, suggesting the presence of universal patterns. Similarly, Jiang et al. [15] used a multi-level meta-analysis to evaluate the impact of biochar on greenhouse gas emissions in CWs, reporting significant effects on CO_2_ mitigation, though, not on CH_4_ and N_2_O emissions. However, these methods may not completely capture the complex interrelationships within datasets, potentially resulting in oversimplified interpretations. This limitation highlights the need for more advanced and robust analytical methodologies.

Artificial intelligence (AI), with its rapid and efficient data processing and analysis capabilities, emerges as a powerful tool for achieving carbon neutrality. Machine learning (ML), a key data-driven modeling technique within AI, excels at handling high-dimensional operational data to extract knowledge, optimize parameters, and mitigate environmental emissions [16,17]. Therefore, ML algorithms offer a promising avenue for predicting CW efficiency because they can learn from large obseravationsand uncover hidden patterns that may be overlooked by traditional modeling approaches [18]. For example, Nguyen et al. [19] successfully predicted the removal of ammonium nitrogen and BOD_5_ in CWs using ML models, including random forests (RFs), generalized linear models, and support vector machines. These approaches may still overlook intricate relationships within the data and lead to oversimplified interpretations, underscoring the need for more sophisticated analytical tools.

**Fig 1:**
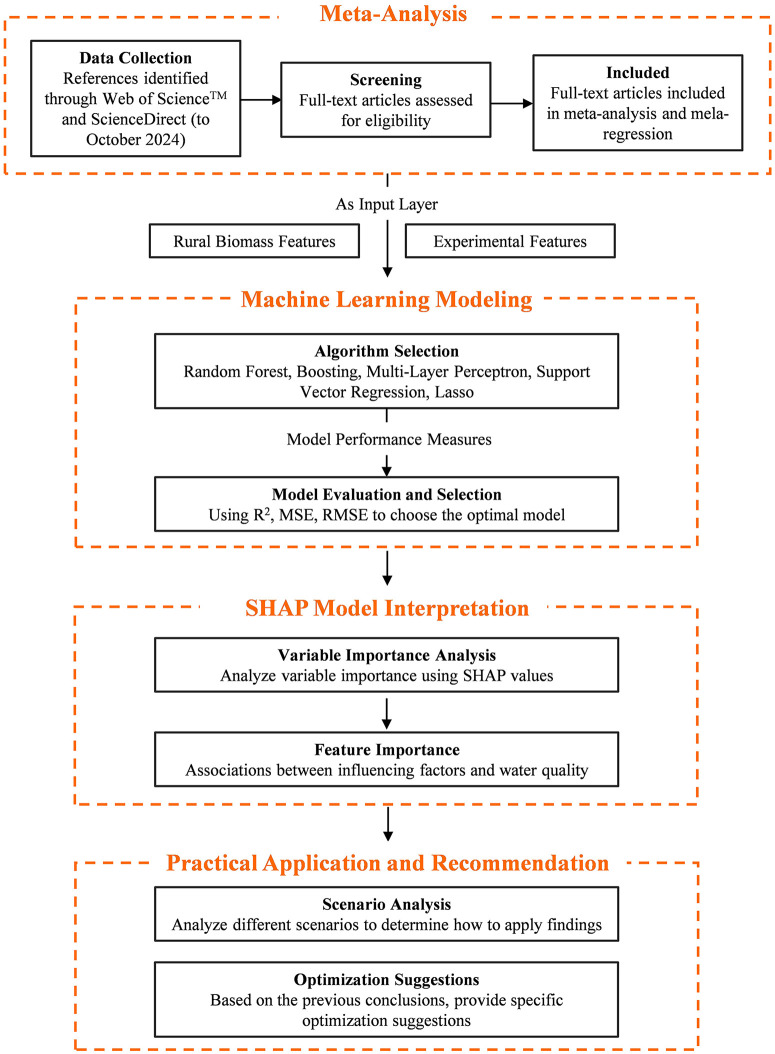
Schematic of the overall workflow.

In this study, we propose an integrated framework that combines meta-analysis, ML, and SHapley Additive exPlanations (SHAP) to address these challenges, is summarized in Fig 1. The research objectives were threefold: (1) to quantify the effects of rural waste amendments on COD and TN removal in CWs via meta-analysis, (2) to identify key influencing factors and predict removal efficiency using ML integrated with SHAP analysis, and (3) to inform the optimization of rural waste applications and CW system design through integrated empirical and computational insights. This approach aims to leverage the strengths of each method, offering a more comprehensive and robust solution for enhancing CW performance and supporting sustainable environmental management.

## Materials and methods

### Literature search and data selection

The dataset on CW performance in treating wastewater with a low C/N ratio was compiled from peer-reviewed papers indexed in the Web of Science (https://www.webofscience.com/) on October 15, 2024. The search strategy employed the following keywords: ((TS=(constructed wetland)) OR (TS=(constructed wetlands)) AND (TS=(low CN)) OR (TS=(low CN ratio)). The time span was limited to the past 20 years. We initially retrieved 91 publications, which were then screened by title and abstract to exclude irrelevant studies. Subsequently, full texts of potentially relevant papers were retrieved and assessed for eligibility according to the following criteria: (1) inclusion of both treatment (agricultural biomass addition) and control (no agricultural biomass addition) groups, and (2) evaluation of the effect of CWs on wastewater treatment, specifically COD and TN removal. Details of the search terms and screening process are provided in SI Text A. After rigorous screening, 70 peer-reviewed publications were included in the meta-analysis, and 15 studies were selected for subsequent ML analysis, Specifically, a total of 272 independent observations were extracted from the 70 studies to construct the meta-analysis dataset. Subsequently, 1,283 high resolution observations were extracted from the 15 selected studies to build the machine learning dataset. A flowchart illustrating the screening process is shown in S1 Fig. This systematic approach ensured that the dataset was robust and representative for investigating the efficacy of CWs in treating low C/N ratio wastewater, in this study low C/N ratio is strictly defined as an influent C/N ratio less than 5.0, as this threshold significantly limits the microbial denitrification process due to carbon source deficiency.

### Meta-analysis

In this study, we employed meta-analysis techniques to evaluate CW performance by focusing on the Normalized Loading Rate (NLR) per unit volume of wetland as a key assessment factor. To achieve this, we compared the NLR across different CW configurations and calculated the effect size to quantify the differences in treatment efficiency. From the screened studies, we extracted three key parameters for the NLR of COD and TN, including the mean NLR values, standard deviations (SD), and number of replicates (n). When standard errors were provided, SD was calculated using the appropriate conversion formula. To estimate the magnitude of the treatment effect, we used the Standardized Mean Difference (SMD) approach, following established methodology [20].


$$\begin{array}{c} NLR(g/m^{\text{3}}\cdot d)=\frac{Q\times C_{influent}-C_{effluent}}{Volume} \end{array}$$

Furthermore, we conducted a K-means clustering analysis of wetland volumes to categorize them into five distinct clusters. K-means clustering was selected because of its simplicity, efficiency, and suitability for largeobservations. This clustering approach enabled the identification of patterns and grouping of similar wetland volumes, providing insights into the relationship between wetland size and treatment performance. The clustering process involved four steps: (1) Data preprocessing—standardizing wetland volume data to avoid bias from scale differences; (2) Determination of optimal clusters—using the elbow method to determine the optimal number of clusters, which was five; (3) Clustering execution—applying the K-means algorithm using the factoextra package in R version 4.1.3 (https://www.r-project.org/), which provided visualizations for interpretation; and (4) Cluster analysis—analyzing each cluster to characterize wetland volumes within each group and their corresponding NLR performance. Meta-analysis was conducted using the metafor package in R, while K-means clustering was performed using the factoextra package, ensuring a comprehensive and statistically robust approach.

### Implementation of ML models

In this study, we employed four supervised ML models—RF [21], Boosting [22], Multi-Layer Perceptron (MLP), and Support Vector Regression (SVR) [18,23]. to simulate the complex relationships between operational parameters and the pollutant removal efficiencies(%) of TN and COD in CWs [24].

To ensure model reliability and prevent data leakage caused by the inherent autocorrelation of multiple observations extracted from the same primary study, we implemented a rigorous study-level grouped nested cross-validation framework. Before model training, data were normalized to address issues related to dimensional inconsistencies and unit differences, thereby enhancing the robustness of the modeling process. Specifically, a nested cross-validation structure was employed: the inner loop was strictly confined to hyperparameter tuning to minimize the root mean squared error (RMSE), while the outer loop was exclusively reserved for evaluating the optimized model on completely untouched hold-out sets. This grouped validation strategy ensures that the models are trained on a subset of studies and evaluated on entirely unseen studies, thereby providing a robust assessment of true external generalization and mitigating the risk of overfitting [25]. Model performance was rigorously evaluated during both calibration and validation phases to prevent overfitting, thereby enhancing the generalizability of the model, particularly with limited training datasets [21]. The caret package in R was utilized to streamline the training, tuning, and validation processes for both the regression and classification tasks, leveraging its integration with numerous ML models. This methodological framework provided a comprehensive and statistically robust approach to understanding the dynamic interactions between operational parameters and pollutant removal efficiency in CWs.

### Model interpretation: ML modeling and SHAP analysis

The model evaluation metrics used in this study were the correlation coefficient (R^2^), mean squared error (MSE), and RMSE. R^2^ quantifies the goodness of fit between the dataset and the regression model, whereas MSE and RMSE, representing the standard deviation of the predicted errors, indicate the dispersion of data points around the regression line.


$$\begin{array}{c} R^{\text{2}}=\text{1}-\left[\frac{\sum_{i=\text{1}}^{n}{\left(y_{i}-x_{i}\right)}^{\text{2}}}{\sum_{i=\text{1}}^{n}{\left(y-\overset{―}{y_{i}}\right)}^{\text{2}}}\right] \end{array}$$

$$\begin{array}{c} MSE=\frac{\text{1}}{n}\sum_{i=\text{1}}^{i=n}{\left(y_{i}-f(x_{i})\right)}^{\text{2}} \end{array}$$

$$\begin{array}{c} RMSE=\sqrt{\sum_{i=\text{1}}^{n}(\frac{{\left(y_{i}-x_{i}\right)}^{\text{2}}}{n})} \end{array}$$

where $f(x_{i})$ is the predicted output value, $y_{i}$ is the true value, $\overset{―}{y_{i}}$ is the average observed value, $x_{i}$ is the predicted value of the algorithm, and n is the number of observations.

Previous studies have often focused on achieving high prediction accuracy while neglecting the interpretability and explainability of the models [26]. Although accuracy metrics may be satisfactory, the lack of interpretability limits the potential insights that can be derived from model predictions. To address this gap, this study employed the SHAP framework, which is based on SHAP values from game theory [27]. SHAP quantifies the contribution of each feature to the prediction outcome and aggregates these contributions to explain the final model output [28]. This method has been widely adopted across various disciplines owing to its ability to provide transparent and interpretable explanations, thereby enhancing the understanding of complex predictive systems [29,30].

In this study, SHAP analysis was employed to provide a detailed interpretation of the predicted results for pollutant removal in CWs [31]. The process involved several steps [32]. Initially, a background distribution was constructed using the training dataset as a reference for evaluating model behavior and feature contributions. Subsequently, various feature coalitions were generated by considering all possible subsets of the selected parameters, including the wetland volume, COD influent concentration, aeration time, aeration rate, influent C/N ratio, TN or COD release from added rural waste, and hydraulic retention time (HRT). For each feature coalition, model predictions were assessed, and SHAP values were calculated. These values represent the average marginal contribution of each feature to model predictions across all possible feature combinations. This method enables a comprehensive understanding of how individual features, such as wetland volume and COD influent concentration, influence the output of the model regarding pollutant removal efficiency. By quantifying the impact of each parameter, SHAP analysis offers robust interpretability and explainability of the prediction results, thereby addressing the gap in previous research, where interpretability was often neglected despite achieving high predictive accuracy [33–35]. This approach not only enhances model transparency but also reveals model inferred feature importance and underlying data patterns associated with pollutant removal in CW systems, which is crucial for advancing the understanding and application of artificial wetland systems in environmental management and decision-making.

Since this study is based on a meta-analysis of previously published literature and computational machine learning modeling, and does not involve human participants or animal subjects, institutional ethical approval and informed consent were not required.

## Results

### Meta-analysis for the waste dataset

**Fig 2:**
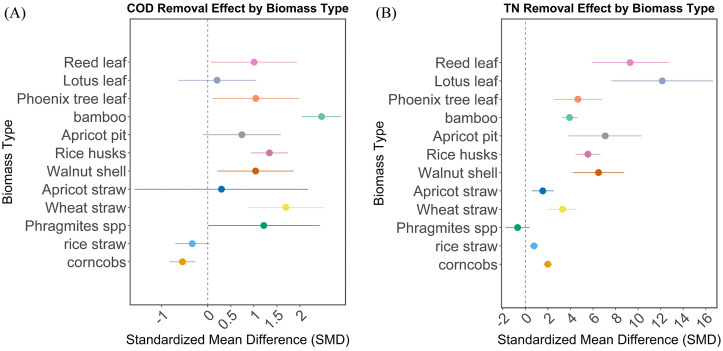
Forest plots of COD and TN removal effects by biomass type. (A) showing the COD removal effect by biomass type, (B) displaying the TN removal effect by biomass type.

As shown in Fig 2A and B, the analysis of different biomass types revealed distinct patterns of effectiveness in COD and TN removal. Bamboo addition produced the greatest enhancement in COD removal, with an SMD of approximately 2.50, indicating a significantly higher contribution than that of the control group (CWs without bamboo addition). For TN removal, lotus leaves demonstrated the most pronounced effect, corresponding to an SMD of approximately 1.2. These SMD values not only quantify the relative performance of different biomass types but also highlight that TN removal efficiency is more responsive to agricultural biomass additions than COD removal. This finding aligns with those of previous experimental studies [36,37], thereby strengthening the credibility of the meta-analysis and underscoring its value in guiding the selection of optimal biomass types for CW applications.

**Fig 3:**
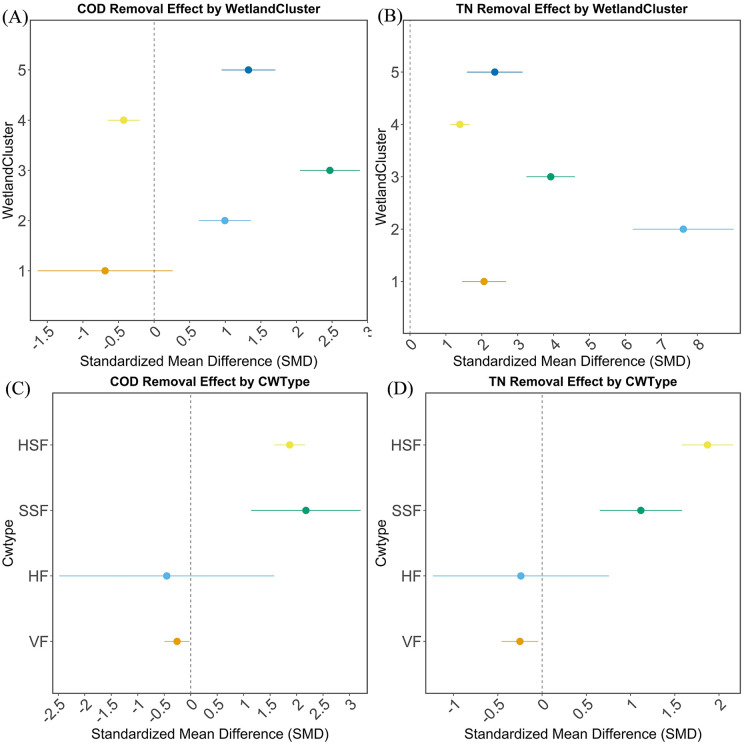
Forest plots of COD and TN removal efficiency by wetland volume and CW type. (A) and (B) show the COD and TN removal effects by wetland volume, (C) and (D) illustrate the COD and TN removal effects by CW type.

This study also explored the influence of different pretreatment methods on the pollutant removal performance of agricultural biomass in CWs. For COD removal, physical pretreatment alone was the least effective approach, whereas the combined physical and chemical pretreatment achieved the highest removal efficiency. A similar pattern was observed for TN removal, where physical pretreatment again showed the least efficiency, while the combined method produced the highest efficiency. As illustrated in S1 Fig, these findings emphasize the importance of pretreatment in maximizing the pollutant removal potential of agricultural biomass. The SMD values linked to these pretreatment methods provide a robust quantitative basis for selecting the most effective pretreatment strategies, thereby enhancing the benefits of biomass addition in CWs, particularly when targeting specific pollutants, such as COD or TN.As shown in Fig 3A and B, wetland volume exerts complex impacts on COD and TN removal efficiencies in CWs. Wetland volume is a critical determinant of pollutant retention and treatment capacity, as larger systems provide more space for biomass and vegetation growth, both of which contribute to pollutant removal. However, the results indicate that increasing wetland volume beyond certain thresholds does not necessarily enhance pollutant removal efficiency. This may be attributed to the saturation of treatment media and microbial communities. When the wetland volume is excessively large, the additional space may not be effectively utilized, as the capacity of the media and microbial communities to degrade or adsorb pollutants can reach a saturation point. Therefore, the optimal wetland volume should be determined by considering both pollutant loading and operational efficiency of the wetland system.

As shown in Fig 3A and b, HRT exerts complex effects on COD and TN removal efficiencies in CWs. HRT is a critical parameter that influences the contact time between pollutants and treatment media, including biomass and wetland vegetation. A sufficient HRT ensures adequate microbial degradation and adsorption. However, the results indicate that merely extending HRT beyond certain thresholds does not guarantee an enhanced pollutant removal efficiency. This is likely due to the saturation of microbial communities and treatment media, where additional time does not proportionally increase the degradation or adsorption capacity [38,39]. Therefore, optimal HRT should balance contact time with the operational efficiency of the wetland system.

Regarding CW type, horizontal subsurface flow (HSF) wetlands demonstrate superior pollutant removal efficiency, particularly when augmented with external carbon sources (Fig 3C, D). HSF systems facilitate uniform distribution of wastewater through the media, enhancing the interaction between pollutants, biomass, and microbial communities. The horizontal flow pattern ensures more uniform contact with the treatment media, improving the utilization of external carbon sources and microbial activity across the wetland. This configuration is particularly advantageous for denitrification and organic matter degradation, which are crucial for efficient COD and TN removal. These findings underscore the importance of selecting both appropriate HRT and CW type to optimize the performance of CWs for pollutant removal.

### Algorithm selection

This study employed several ML models to predict the COD removal efficiency of CWs. To coherently integrate the findings from the meta-analysis, the categorical variations in biomass types were quantitatively translated into continuous physical and chemical features, specifically ‘biochar COD release’ and ‘biochar TN release,’ allowing the ML models to capture the underlying material driven mechanisms. The input features included C/N ratio, wetland volume, HRT, aeration time, aeration rate, biochar COD release, biochar TN release, and influent COD concentration. These input features were selected based on the high relevance factors identified in a previous meta-analysis and findings of prior studies [40,41]. The output variable was the COD removal efficiency of CWs.

**Fig 4:**
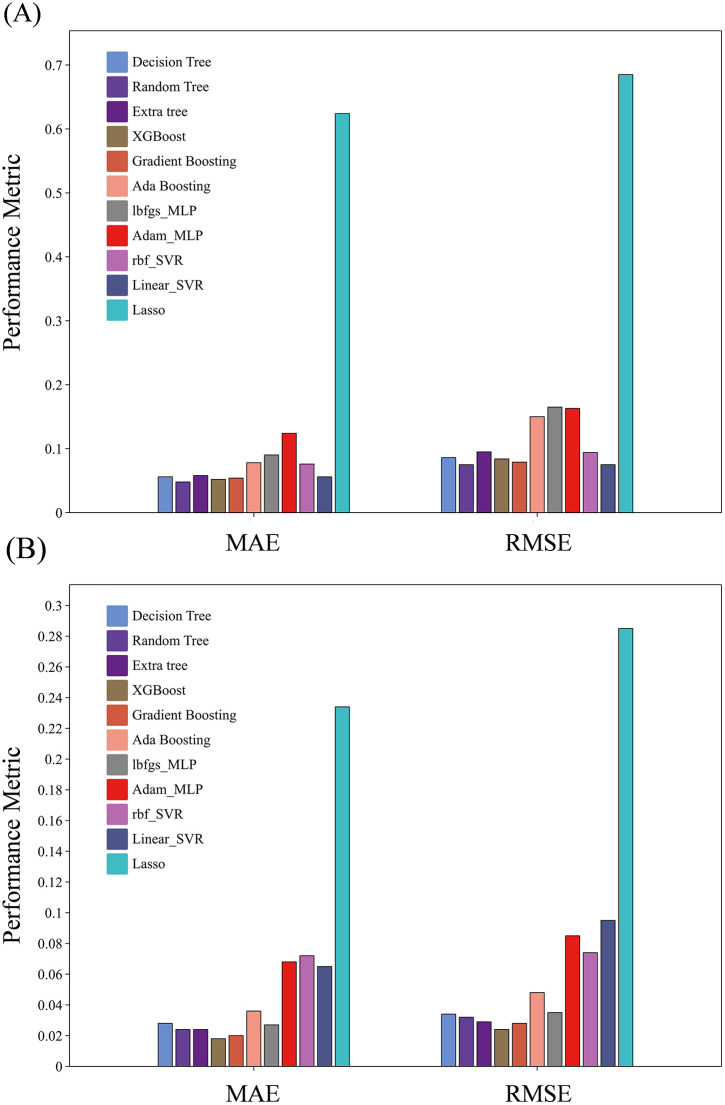
Comparison of machine learning models’ performance in predicting COD removal efficiency in constructed wetlands. (A) bar charts comparing the MAE and RMSE of different machine learning models in predicting COD removal efficiency (B) bar charts comparing the MAE and RMSE of different ML models in predicting TN removal efficiency.

S2 Fig presents the scatter plots of actual versus predicted COD removal efficiencies for each model, with corresponding R^2^ values to quantify model performance. Fig 4A shows bar charts of MAE and RMSE for each model, providing a visual comparison of model accuracy. The scatter plots in S2 Fig reveal that the RF model exhibited the highest R^2^ value (0.76), demonstrating reliable prediction accuracy. The Lasso model recorded the lowest R^2^ value (0.29), indicating inadequate performance for capturing the intricate dynamics of COD removal in CWs. This discrepancy highlights the need for nonlinear models, such as MLP, RF, and SVR, to accurately predict COD removal efficiency. The bar charts in S2 Fig show that the Lasso model had the highest MAE (0.624) and RMSE (0.685), indicating poor predictive accuracy and reliability. This suggests that the relationship between the input features and COD removal efficiency is not adequately captured by a simple linear model. Conversely, the RF model demonstrated satisfactory performance, with an R^2^ of 0.76, MAE of 0.048, and RMSE of 0.075, indicating its reliable ability to model complex nonlinear relationships in the data.

Several ML models were employed to predict the TN removal efficiency of CWs. The input features were similar to those used in the COD removal efficiency prediction, except that the influent TN concentration was used instead of the influent COD concentration. S3 Fig presents scatter plots of actual versus predicted TN removal efficiencies for each model, with R^2^ values displayed in each subplot to quantify performance. It also shows bar charts of the MAE and RMSE for each model, offering a visual comparison of the model accuracy. The bar charts in Fig 4B indicate that the Lasso model again exhibits the highest MAE and RMSE, showing poor prediction accuracy and reliability. This finding reinforces the idea that the relationship between input features and TN removal efficiency is not adequately captured by a simple linear model. In contrast, the XGBoost model demonstrated effective mechanisms performance, with an R^2^ of0.82, MAE of0.018, and RMSE of0.024, indicating its satisfactoryability to model complex nonlinear relationships in the data. The scatter plots in S3 Fig further demonstrate the high accuracy of XGBoost, which consistently outperformed other models. Similar to the COD removal analysis, the Lasso model achieved the lowest R^2^, further underscoring its poor performance in capturing the intricate dynamics of TN removal in CWs. These findings highlight the necessity of employing nonlinear models, such as XGBoost, MLP, RF, and SVR, to accurately predict TN removal efficiency.

When comparing the overall performance of the models predicting TN removal efficiency and those predicting COD removal efficiency, the TN models consistently achieved higher prediction accuracy. The MAE and RMSE values for the TN models were generally lower than those for the COD models, indicating that the models were better at capturing nonlinear relationships in TN removal. This finding suggests that the input features might have a more defined and predictable relationship with TN removal than with COD removal, or that the TN removal process in CWs is more amenable to modeling with the selected features and algorithms. The reliableperformance of the XGBoost model in TN removal prediction, with its low MAE and RMSE, makes it the most suitable model for predicting TN removal efficiency in CWs. This finding underscores the importance of selecting appropriate models that can effectively handle data complexity and the nonlinear nature of the processes involved in pollutant removal in CWs.

### Model interpretation by SHAP analysis

**Fig 5:**
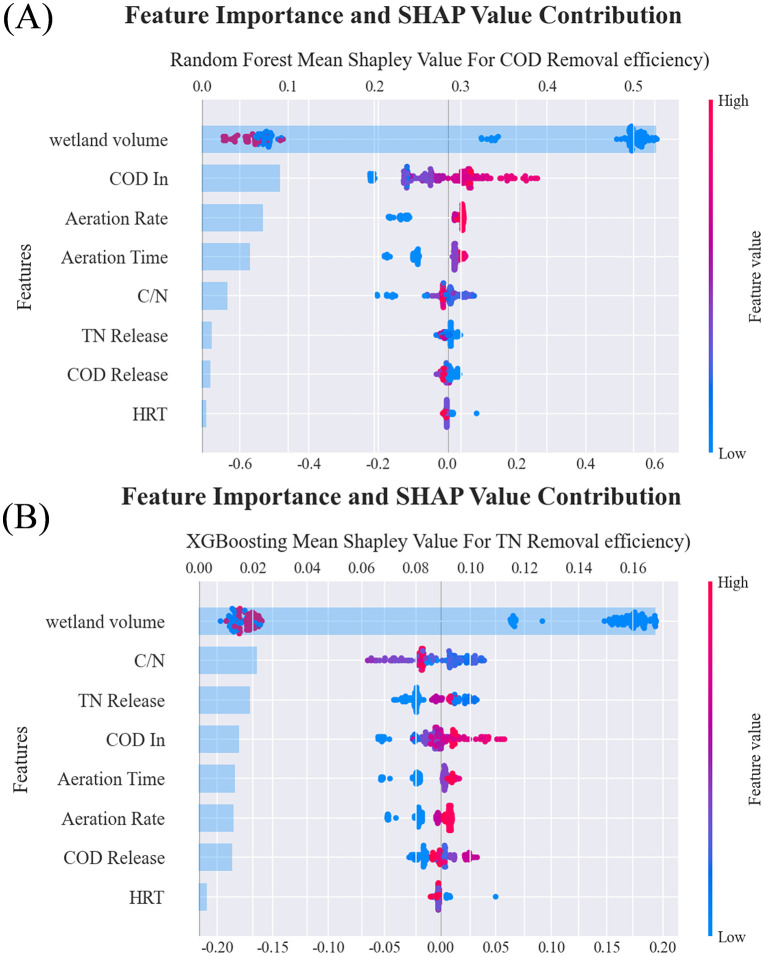
Feature importance and SHAP value contributions for COD and TN removal efficiency prediction in constructed wetlands. Subfigure (A) illustrates the feature importance and SHAP value contribution for predicting COD removal efficiency using the Random Forest model, demonstrating the relative significance of various features through their mean SHAP values, while (B) presents the same for predicting TN removal efficiency using the XGBoost model, highlighting the key features influencing the model's predictions by displaying their respective SHAP values.

SHAP analysis was performed using the RF model, which was identified in a previous study as the optimal model for simulating COD removal efficiency in CWs. The bar chart illustrates the mean SHAP value for each feature, indicating its relative importance in the model as shown in Fig 5. Longer bars indicate a greater influence of the feature on the model's predictions. The three most influential factors were wetland volume, COD influent concentration, and aeration rate. This indicates that, while the addition of rural waste as an external carbon source does not significantly enhance COD removal efficiency, increasing aeration may be more beneficial for improving COD removal in CWs. These results align with those of previous studies, confirming the reliability of the findings.

The scatter plot displays SHAP values against feature values, with a color gradient representing feature values. This visualization highlights how variations in feature values influence model predictions, revealing both the direction and magnitude of their impact on COD removal efficiency. These insights provide a valuable basis for optimizing CW operations to enhance COD removal.

Focusing on the scatter plot, wetland volume showed a positive correlation with COD removal efficiency, indicating that larger volumes generally contribute to higher removal rates. This is likely due to the increased surface area and longer HRT provided by larger wetlands, allowing for more extensive treatment processes. In contrast, TN influent concentration exhibited a negative correlation with COD removal efficiency. Higher TN concentrations may compete with COD for degradation pathways or reflect more complex wastewater compositions that are difficult to treat, suggesting that while TN itself is not directly removed in this context, its presence can influence COD removal dynamics. These detailed insights clarify how specific features drive model predictions and can inform strategies to optimize CW design and operation.

SHAP analysis revealed a predominantly positive correlation between aeration rate and COD removal efficiency. Higher aeration rates generally enhanced COD removal, as indicated by positive SHAP values associated with this feature. The scatter plot highlights an optimal range of aeration rates where COD removal efficiency is maximized. Beyond this range, further increases in aeration may not yield proportional improvements in COD removal and could lead to diminishing returns or even negative effects. The impact of aeration rate is also influenced by interactions with other features. For instance, higher aeration rates combined with sufficient wetland volume and appropriate C/N ratios create synergistic effects that further enhance COD removal efficiency.

The addition of biochar in CWs can effectively enhance COD removal efficiency. This improvement can be attributed to biochar’s porous structure and large specific surface area, which enable physical adsorption and filtration of organic matter in wastewater. Additionally, functional groups on the biochar surface can interact with organic matter through electrostatic attraction or repulsion and form hydrogen bonds with organic compounds. Biochar also provides a favorable habitat for microorganisms, promoting their growth and activity, which facilitates the biodegradation of organic matter.

SHAP analysis was conducted using the XGBoost model, which was identified in a previous study as the optimal model for simulating TN removal efficiency in CWs. The bar chart in the figure represents the mean SHAP value for each feature, indicating their relative importance in the model. The three most influential factors were the wetland volume, TN influent concentration, and TN release from the added carbon source. This highlights the important role of external carbon sources, such as biochar, in enhancing TN removal efficiency in CWs.

Focusing on the scatter plot, wetland volume showed a positive correlation with TN removal efficiency, suggesting that larger systems generally contribute to higher removal rates. This is likely due to the increased surface area and longer HRT provided by larger wetlands, allowing for more extensive treatment processes. For TN influent concentration, the scatter plot revealed a complex nonlinear relationship with TN removal efficiency. Higher TN concentrations may initially enhance removal rates because of increased microbial activity, however, may eventually saturate the system, leading to diminishing returns. The TN released from the added carbon source positively contributed to TN removal efficiency, suggesting that the addition of external carbon sources, such as biochar, provides additional adsorption sites and promotes microbial degradation of TN, thereby enhancing TN removal efficiency.

After adding biochar to CWs, TN removal efficiency exceeded that of COD. Specifically, certain bacteria are involved in the nitrification process, converting ammonia nitrogen into nitrate nitrogen, whereas others utilize organic carbon sources to reduce nitrate to nitrogen gas during the denitrification process. The introduced biochar provides an appropriate habitat along with sufficient organic carbon sources to support the growth and metabolic activities of these microorganisms, thereby enhancing TN removal. In contrast, COD removal predominantly depends on the adsorption and degradation of organic matter by the biochar and microorganisms. The complexity and variability of organic matter present in wastewater may result in a relatively lower COD removal efficiency than TN removal efficiency.

## Discussion

### Effectiveness of the proposed framework

This study proposes an integrated analytical framework that combines meta-analysisand interpretable ML to evaluate the influence of agricultural biomass addition on pollutant removal in CWs, while aligning data-driven feature insights with established ecological principles. Unlike previous CW studies that primarily focused on site-specific performance or single-factor optimization, this approach provides both predictive accuracy and mechanistic insight, supporting its application across diverse ecological and operational contexts.

As highlighted in the Introduction, traditional CW studies often suffer from site-specific limitations, limited reproducibility, and a lack of generalizable design principles [12]. Prior studies have demonstrated the predictive capacity of ML; for instance, ANN models achieved R^2^ values up to 0.835 for TN removal in low-OLR horizontal flow CWs [36], and SVM models reached an R^2^ of 0.736 for predicting total phosphorus removal in hybrid CWs under variable meteorological and hydraulic conditions [42]. However, these approaches typically focus on specific pollutant indicators and lack interpretability or integration across system components.

Recently, a literature-data-statistics framework was developed to identify the key factors influencing antibiotic and ARG removal in CWs, highlighting elements, such as mobile genetic elements, plant types, and hydraulic conditions [43]. While these studies offer valuable insights, they primarily focus on either descriptive factor analysis or pollutant-specific system optimization.

Building on these developments, our framework extends the analytical depth by incorporating both global performance synthesis via meta-analysis and interpretable predictions via ML and SHAP. The meta-analysis qualitatively identified the optimal biomass materials, while the ML models, utilizing the carbon release features derived from these materials, quantitatively predicted their performance under complex operational matrices. This approach not only achieved high predictive accuracy(R^2^ > 70for COD and TN) but also revealed the influence of specific features such as biomass type, wetland volume, and aeration rate. This dual capacity positions the framework as both a predictive engine and a transparent decision support tool for optimizing CW design, operation, and resource reuse.

### Agricultural biomass additions enhance pollution removal efficiency in CWs

The addition of agricultural biomass, particularly biochar, enhances pollutant removal in CWs through multiple interacting ecological processes. These include: (1) providing labile and recalcitrant carbon sources to support denitrifying microbial communities, (2) increasing surface area and porosity for microbial colonization and organic matter adsorption, and (3) modifying redox gradients to facilitate coupled nitrification–denitrification pathways [6].

Our results demonstrated that TN removal efficiency consistently exceeds that of COD removal in biochar-amended CWs. This is attributed to the enrichment of functional microorganisms, such as nitrifying and denitrifying bacteria, which are supported by the porous structure and surface chemistry of biochar [11,44]. These microbial communities catalyze nitrogen transformation processes, such as ammonia oxidation and nitrate reduction, leading to efficient TN removal, even under low C/N conditions. In contrast, COD removal is more dependent on biochar’s physical adsorption and microbial degradation of complex organic compounds, which may vary in solubility and degradability. When the amount of biochar is reduced, COD removal efficiency declines sharply, underscoring the importance of maintaining sufficient reactive surfaces for adsorption and microbial colonization [45–47].

Moreover, aeration played a critical role in COD removal. Although increased aeration generally enhances oxygen availability and organic degradation, our results indicate that excessive aeration under short HRT conditions can lead to suboptimal removal owing to insufficient contact time [48]. Therefore, achieving synergistic improvements requires balancing optimal aeration intensity with appropriate wetland volume and HRT.

Collectively, these findings reinforce the concept discussed in the Introduction: agricultural biomass not only serves as a low-cost external carbon source but also contributes to the ecological and operational sustainability of CWs, aligning with circular economy principles and offering a viable pathway for waste valorization and rural water treatment co-optimization.

### Limitations and future perspectives

This study had a few limitations. First, while the framework demonstrated strong predictive capability and mechanistic insight, the dataset was limited by the availability of studies reporting both biomass properties and detailed operational parameters. Therefore, expanding the range of biomass types and pretreatment methods in future studies will strengthen the ecological generalizability of the findings. Second, the sample size may be insufficient, as only 70 of 91 manually screened articles were included in the meta-analysis and 15 in the ML models, which may not completely represent the breadth of research in this field, and the literature search was restricted to the Web of Science Core Collection. This single-database approach and the specific keyword set employed may introduce potential retrieval biases, potentially omitting relevant studies indexed in other databases. Finally, the manual article screening process lacked reverse validation. Specifically, this study did not use SHAP-derived feature contributions to verify laboratory experiments, which could have provided additional insights into the relationships between variables.

Future research should further explore the use of rural waste-derived biochar as an external amendment to enhance pollutant removal efficiency in CWs. Additionally, further studies should expand the range of rural waste types used as external carbon sources to help identify more efficient and cost-effective materials for pollutant removal and contribute to waste recycling and resource utilization. Beyond the percentage of pollutant removal in CWs, future studies should focus on efficiency, such as the rate of pollutant removal per unit time and volume, which can provide a more accurate and practical measure of the pollutant removal performance in CWs. Moreover, this study revealed that appropriately increasing the volume of CWs could improve their pollutant removal efficiency to a certain extent. Therefore, future research should investigate the optimal design and operational conditions of CWs to maximize pollutant removal while considering factors, such as cost and space requirements.

## Conclusions

This study presented an integrated framework that combines meta-analysis and ML to evaluate the impact of agricultural biomass addition on pollution removal efficiency in CWs. The meta-analysis revealed that bamboo biochar improved COD removal efficiency by approximately 54.8%, whereas lotus leaf biochar enhanced TN removal efficiency by approximately 32.5% compared with controls. Wetland volume, influent concentration, and aeration rate were identified as the dominant ecological and operational drivers. These findings align with the established premise that biomass addition improves CW performance through multiple mechanisms, including enhanced microbial colonization, increased adsorption capacity, and optimized redox gradients that facilitate coupled nitrification–denitrification.

From an ecological engineering perspective, these results highlight the need to align biomass type with target pollutants, optimize hydrological retention through appropriate wetland volume, and balance aeration to sustain diverse microbial pathways. Valorizing rural agricultural waste as a functional component of CWs supports the circular economy, reduces reliance on synthetic inputs, and enhances the multifunctionality of CWs as nature-based solutions for water quality improvement, biodiversity support, and climate resilience.

Future research should integrate large-scale field validation with modeling frameworks to ensure the robustness of predicted ecological responses under varying climatic and hydrological regimes, thereby advancing transferable design principles for global CW applications.

## Supporting information

S1 Fig Forest plots of COD and TN removal efficiency by HRT, CW Type, CW Volume and pretrement. (JPG)

S2 Fig Comparison of machine learning models’ performance in predicting COD removal efficiency in constructed wetlands. (PNG)

S3 Fig Comparison of machine learning models’ performance in predicting TN removal efficiency in constructed wetlands. (PNG)
