# Supplementary figures and images for: Pollution removal efficiency enhancement by agricultural biomass additions in constructed wetlands: A framework integrating meta-analysis with explainable machine learning
Source: PLoS One. 2026 Jul 7;21(7):e0353064. doi: 10.1371/journal.pone.0353064 (PMC13340836; doi:10.1371/journal.pone.0353064)

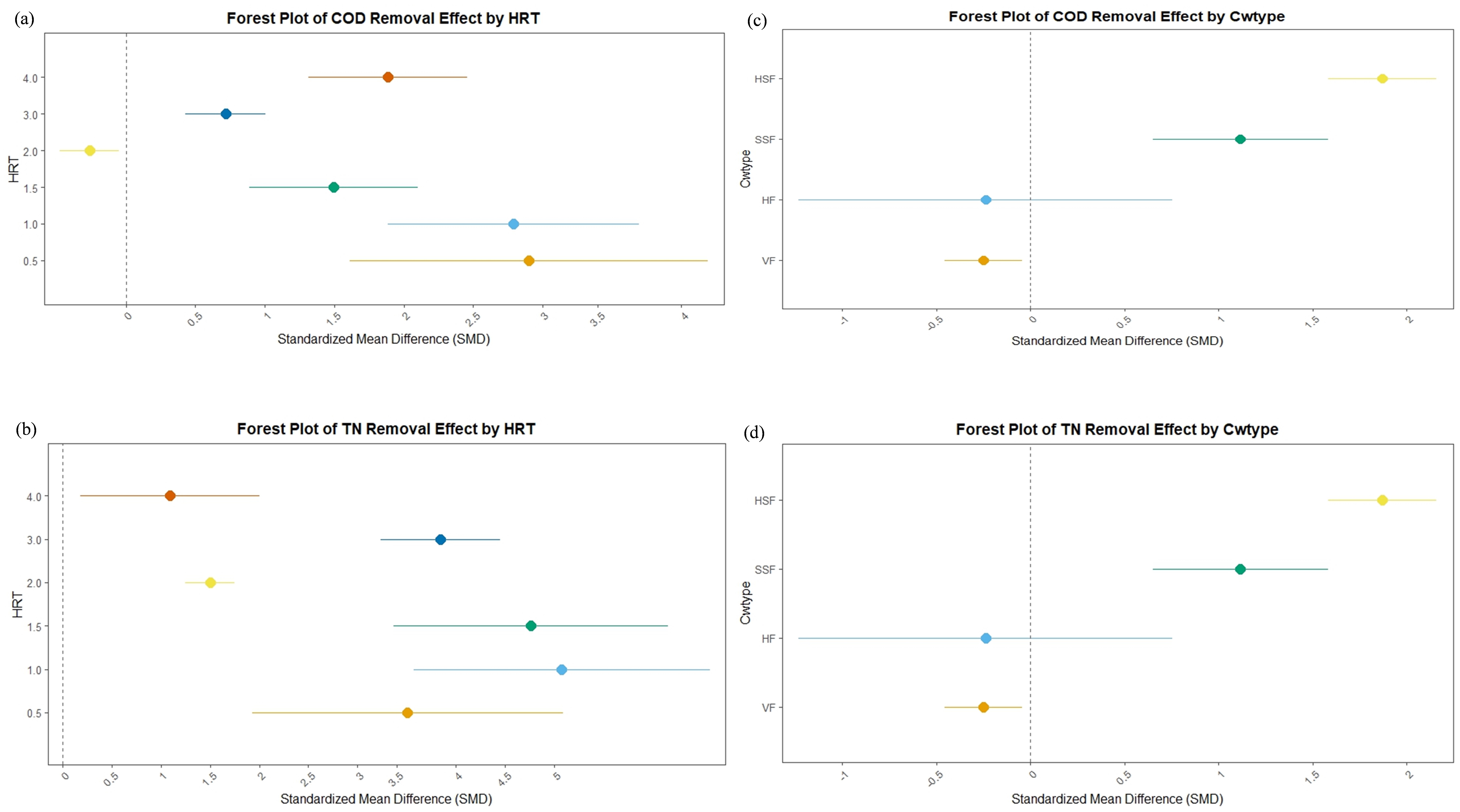

Supplement: S1 Fig — (JPG) [file pone.0353064.s001.jpg]

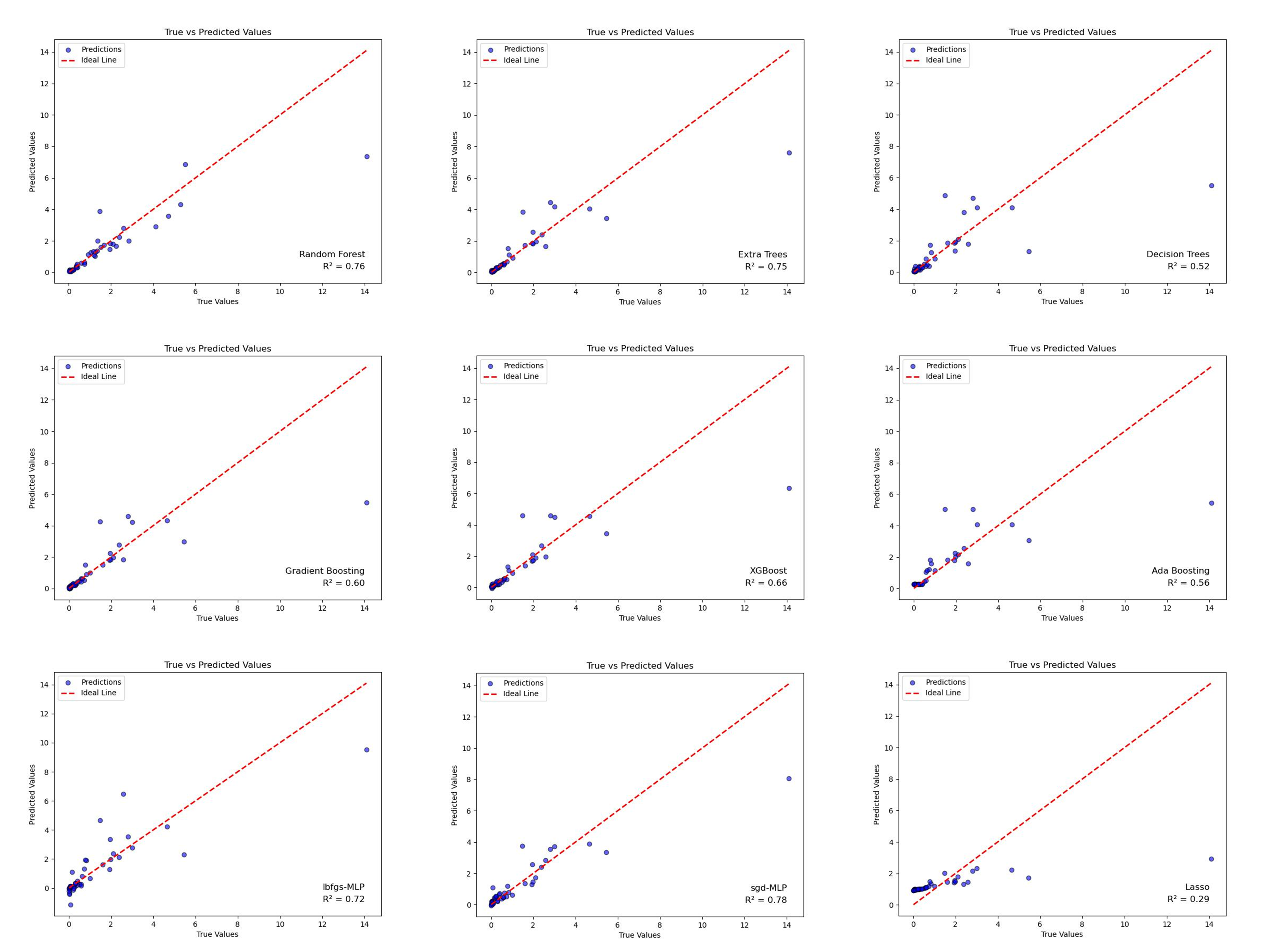

Supplement: S2 Fig — (PNG) [file pone.0353064.s002.png]

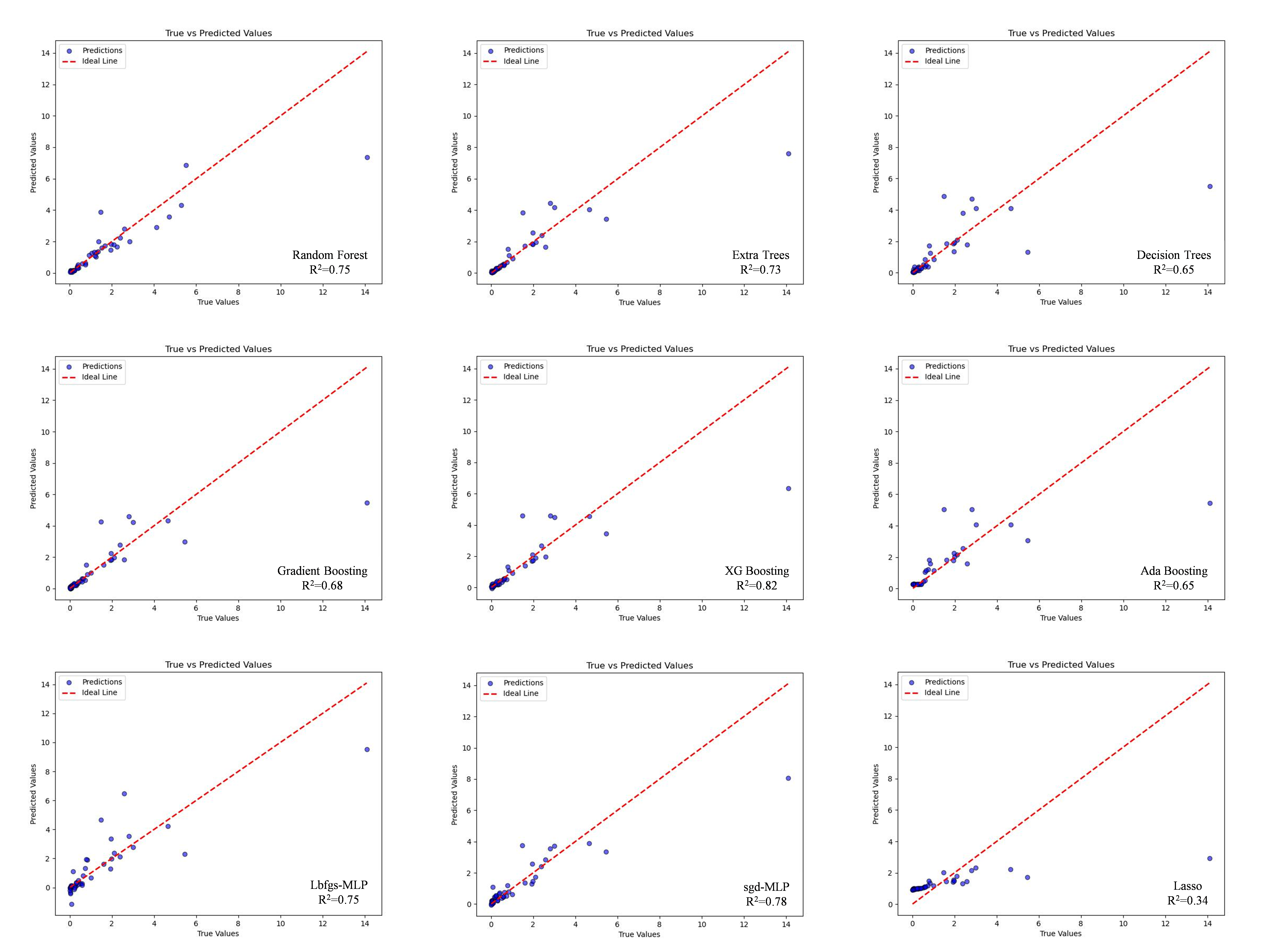

Supplement: S3 Fig — (PNG) [file pone.0353064.s003.png]
