# Supplementary material for: Pollution removal efficiency enhancement by agricultural biomass additions in constructed wetlands: A framework integrating meta-analysis with explainable machine learning
Source: PLoS One. 2026 Jul 7;21(7):e0353064. doi: 10.1371/journal.pone.0353064 (PMC13340836; doi:10.1371/journal.pone.0353064)
Supplement: Attachment — Submitted filename: Response to Reviews.docx [file pone.0353064.s005.docx]

March 27^th^, 2026

Dear Editor and Reviewers:

Enclosed please find the revised manuscript (PONE-D-26-02227), “Pollution Removal Efficiency Enhancement by Agricultural Biomass Additions in Constructed Wetlands: A Framework Integrating Meta-Analysis with Explainable Machine Learning” by Wenjie Li, Jian Wang, Xinya Liu, Yulin Zhuang, Hongda Fang, Jinliang Huang. We appreciate the time and efforts you and the reviewers have dedicated to providing valuable feedback on our manuscript. We have ensured that each comment has been carefully addressed and the revised manuscript is now clearer and stronger. We are now submitting it for further consideration for publication in your esteemed journal. Please note that the line number in each response corresponds to the line number in the revised manuscript with marked changes.

**Updated Funding Statement:**

This research was supported by the Natation Natural Science Foundation of China (Grant No.42376225). The funders had no role in study design, data collection and analysis, decision to publish, or preparation of the manuscript.

**Data Availability:**

All data and author-generated code supporting the findings of this study are publicly available on GitHub at: https://github.com/leeeee-max/Plos-One.git. There are no restrictions on access.

**Reviewer #1:**

**General comments:**

Your manuscript tackles a genuinely worthwhile idea regarding using agricultural biomass in constructed wetlands to improve performance under low C/N conditions, and the “meta-analysis+explainable ML” framing could have been a strong contribution. Unfortunately, the paper in its current form reads like two separate studies stapled together, with major inconsistencies in definitions, equations, and what exactly is being predicted.

**Response:** We sincerely appreciate your positive comments. We have thoroughly revised the manuscript to improve its clarity, conciseness, and overall academic writing quality. We fully agree with your assessment and have implemented a comprehensive restructuring of the manuscript to explicitly demonstrate that this is a single, organically integrated framework designed to comprehensively answer our core scientific question: How can agricultural biomass addition optimize pollution removal in constructed wetlands under low C/N conditions? To clarify the indispensable connection between the two parts, we have revised the manuscript and structured our integration logic.

1. There is a direct contradiction between the methods section, which claims to use a log-transformed response ratio, and the results section, which reports Standardized Mean Difference (SMD). The metric must be uniform, clearly defined, and justified.

**Response:** Thank you for your suggestion. To clarify, the actual statistical metric calculated and utilized throughout our analysis was the Standardized Mean Difference (SMD), specifically applying the Hedges' g correction. The mention of the "log-transformed response ratio" in the original Methods section was an oversight during the manuscript drafting process and did not reflect the actual data pipeline we executed. We have now comprehensively revised the manuscript to ensure absolute uniformity. All references to the response ratio have been removed, and we have explicitly defined and justified our use of SMD in the revised Section 2.2 (Meta-Analysis). We have provided the exact computation formulas for the SMD and its variance estimation to ensure full transparency and reproducibility. We deeply appreciate the your meticulous reading, which has significantly tightened the methodological rigor of our paper.

2. The formula used for Nitrogen Loading Rate (NLR) is mathematically and physically flawed. Including both wetland volume and Hydraulic Retention Time (HRT) in the denominator causes redundant normalization (since HRT is already volume-dependent), compromising the validity of the downstream effect sizes.

**Response:** Thank you for your constructive suggestion. We have corrected the formula in the revised manuscript (Section 2.2). More importantly, regarding your highly valid concern about the validity of the downstream effect sizes and our machine learning models, we would like to explicitly clarify that this flawed equation was strictly a typographical and drafting error in the manuscript text, rather than a systematic flaw in our analytical pipeline. During our actual data extraction and computational processing, the mass removal rates were correctly computed based on the influent flow rate ($Q$) and the actual wetland volume, strictly avoiding the redundant incorporation of HRT. We have thoroughly re-audited our datasets and code scripts to ensure complete dimensional accuracy. We confirm that all computed effect sizes in the meta-analysis, as well as the input features for the machine learning models, were based on this correct physical logic. Therefore, the downstream effect sizes, along with the SHAP interpretations highlighting the importance of wetland volume, remain entirely robust and unaffected by this textual oversight. We deeply appreciate your rigorous mathematical checking, which has helped us eliminate this confusing typo and ensure the technical soundness of the paper.

3. The methodology for study selection lacks transparency and reproducibility. Specifically, the Boolean search string has operator precedence issues, and the threshold/definition for "low C/N" (a foundational premise of the study) is not explicitly defined.

**Response:** We sincerely appreciate the reviewer's meticulous evaluation of our methodology. We have completely addressed both issues in the revised manuscript. Following your highly constructive advice, we have now explicitly defined this threshold and its biogeochemical justification in Section 2.1. Specifically, we have added the statement to the revised manuscript. We believe these revisions have fundamentally improved the transparency, reproducibility, and scientific rigor of our study selection process. Thank you again for pointing out these methodological gaps.

4. Reporting unusually high R^2^ values (0.98–0.99) on heterogeneous environmental data strongly suggests data leakage or overfitting. The manuscript fails to clearly separate training, hyperparameter tuning, and out-of-sample validation (e.g., nested cross-validation).

**Response:** Thank you for your suggestion. We are deeply grateful to the reviewer for this sharp and highly critical observation. We fully agree that the initial R^2^ values of 0.98 were a clear red flag. Upon rigorous re-evaluation, we identified that this artificially inflated performance was caused by a combination of mathematical coupling in the feature space and data leakage stemming from standard random data splitting across clustered literature datasets. To address this fundamental methodological flaw, we have completely overhauled our machine learning pipeline and implemented a rigorous, leakage-free validation strategy in the revised manuscript. We have abandoned the previous ambiguous train-validation split. Instead, we established a strict nested cross-validation framework. Hyperparameter tuning is now strictly confined to the inner cross-validation loop. The optimized model is then evaluated exclusively on the completely untouched outer loop hold-out sets. This guarantees an absolute separation between model tuning and out-of-sample validation. To prevent data leakage where observations from the same primary study appear in both the training and testing sets, we replaced simple random splitting with Study-Level Grouped Cross-Validation. The models are now trained on a subset of studies and tested on entirely unseen studies, ensuring that the validation metrics reflect true external generalization. We have updated the Results section (Section 3.2), the Abstract, and the relevant Figures to reflect these new metrics. Furthermore, we have completely removed exaggerated claims such as "optimal" or "outstanding" from the text. We sincerely thank the reviewer for guiding us to significantly improve the rigor of our computational framework.

5. The prediction target is inconsistently described (removal efficiency vs. first-order rate constant k). More critically, the input features include variables mathematically entangled with the target. This means the model might simply be learning a rearranged mathematical identity rather than discovering genuine predictive relationships (a tautology).

**Response:** We are deeply grateful to the reviewer for this highly professional and critical observation. To completely resolve this issue and ensure the scientific validity of our ML models, we have implemented a comprehensive overhaul of Section 2.3 and the corresponding Results section. We have removed all equations and mentions regarding the prediction of the first-order areal rate constant. In the revised manuscript, we explicitly clarify that the sole prediction targets for our machine learning models are the absolute TN and COD Removal Efficiencies (%). This strategic decoupling forces the XGBoost and Random Forest algorithms to genuinely learn the underlying non-linear biogeochemical relationships and treatment dynamics, rather than merely reverse-engineering an algebraic identity. Consequently, the SHAP analysis now provides defensible, mechanistic insights into how specific operational drivers causally influence the overall pollutant removal performance. We believe these critical modifications have fundamentally strengthened the robustness and interpretability of our machine learning framework.

6. The SHAP analysis currently reads as a causal or mechanistic explanation of the physical system, rather than what it actually is: an explanation of the model. Because the input features may be proxies for the target, the SHAP results lack validity without strict robustness checks and external validation.

**Response:** We deeply appreciate this profound methodological and philosophical critique. We fully agree with your assessment. It is a common pitfall in applied machine learning to conflate model-inferred feature importance with true physical causality. SHAP strictly explains how the model reaches its specific predictions based on the given data, and we agree that it should not be overstated as a direct proof of biogeochemical mechanisms. To rigorously address this crucial point, we have implemented a comprehensive revision in our manuscript (specifically in Sections 2.4, 3.3, and 4). We have meticulously revised the language throughout the manuscript to remove any deterministic causal claims. Phrases such as "reveals the mechanism" or "drives the removal" have been replaced. Instead, we now explicitly state that SHAP is utilized for revealing the underlying data pattern and model-inferred feature interpretation. We have clarified that while SHAP explains the model's behavior, we only use it to observe whether the model's logic aligns with established ecological principles, rather than claiming it discovers them. Regarding your highly valid concern that input features might simply be proxies for the target, this issue was fundamentally resolved by our structural revision (as detailed in our response to your previous comments regarding mathematical coupling). By shifting the prediction target exclusively to the absolute Removal Efficiency and eliminating all mathematically coupled variables, our input features are now strictly independent operational and environmental parameters. They are no longer mathematical proxies for the target, ensuring that the SHAP values reflect genuine operational influence rather than tautological relationships.

7. The manuscript promises a unified framework but delivers two disconnected pipelines. The key findings from the meta-analysis (e.g., optimal biomass types) are not coherently integrated into the feature engineering or the interpretations of the machine learning model.

**Response:** We are extremely grateful for this insightful critique. We fully agree that in the original manuscript, the narrative bridge between the meta-analysis findings and the machine learning (ML) feature engineering was not articulated clearly, giving the unintended impression of two disconnected pipelines. We have completely rewritten the transition between these two modules (Section 3.2 and Section 4.1) to explicitly demonstrate how they form a single, coherent, and sequential framework. You rightfully pointed out that if "biomass type" is the major conclusion of the meta-analysis, it should be reflected in the ML features. In the revised Section 3.2 (Feature Engineering), we now explicitly explain our feature selection rationale: rather than feeding categorical labels (e.g., "bamboo" or "lotus leaf") into the XGBoost model, which limits generalizability to unseen materials, we quantitatively translated the optimal traits identified in the meta-analysis into continuous operational features. Specifically, the distinct physicochemical properties of different biomass types were engineered into the features "biochar COD release" and "biochar TN release". This represents a direct, coherent data bridge: the meta-analysis identified which materials work best qualitatively, and the ML feature engineering captured why they work quantitatively. In the revised Discussion (Section 4.1), we have harmonized the interpretation. The SHAP analysis revealed that "TN release from the added carbon source" is a top-three influential feature. We now explicitly discuss how this ML finding perfectly corroborates the meta-analysis finding: the superiority of lotus leaf and bamboo biochar (found in the meta-analysis) is mathematically driven by their specific carbon/nitrogen release kinetics and highly porous structures (validated by the ML/SHAP analysis).

**Reviewer #2:**

1. The manuscript uses the terms datasets, studies, and observations inconsistently. The Abstract reports 272 and 1,283 datasets for meta-analysis and machine learning, whereas Section 2.1 states that 70 studies were used for meta-analysis and 15 studies for ML. The relationship between these numbers is not defined. The authors should clearly explain what constitutes a dataset, how many observations were extracted per study, and ensure consistent terminology throughout the manuscript.

**Response:** We sincerely thank the reviewer for pointing out this critical issue regarding terminology inconsistency, and we apologize for the confusion caused by our previous misuse of the word "datasets" to refer to individual "observations." We fully agree that precise data terminology is essential for the reproducibility and clarity of this framework. To address your concern, we have completely standardized our terminology throughout the revised manuscript (specifically in the Abstract and Section 2.1) by strictly defining a "study" as an independent, peer-reviewed publication retrieved from our literature search, an "observation" as a single experimental measurement extracted from a study, and a "dataset" as the entire compiled matrix of observations used to execute a specific analytical algorithm. Based on these strict definitions, we have clarified the relationships between the reported numbers: for the meta-analysis, we screened 70 studies to extract a total of 272 independent observations (averaging approximately 3.9 observations per study), which collectively constitute our "meta-analysis dataset." For the machine learning (ML) analysis, we selected a subset of 15 studies from this pool because they reported the highly detailed, high-dimensional operational parameters required by ML models. Consequently, we were able to extract a substantial 1,283 independent observations from just these 15 studies (averaging approximately 85.5 observations per study) to form our "ML dataset." Accordingly, we have corrected the Abstract to state "...using 272 and 1,283 independent observations extracted from 70 and 15 published studies for meta-analysis and machine learning, respectively," and we have explicitly added a paragraph in Section 2.1 defining these relationships and the average number of observations extracted per study. We believe these corrections completely remove any ambiguous use of the word "datasets," eliminating previous contradictions and significantly improving the rigorousness of our data reporting. Thank you again for your meticulous reading.

2. Section 2.2 states that a log-transformed response ratio was used to calculate effect sizes, but the Results section reports standardized mean difference (SMD) values. These are different statistical measures and cannot be used interchangeably. The authors must clarify which effect size metric was actually applied and revise either the Methods or Results so that the reported outcomes align with the stated methodology.

**Response:** We sincerely apologize for this discrepancy and thank the reviewer for their careful reading. As also pointed out by Reviewer #1, this was a typographical and drafting oversight in the original manuscript's Methods section. We can unequivocally confirm that the actual statistical metric calculated and utilized throughout our data processing and meta-analysis was the Standardized Mean Difference (SMD), specifically applying the Hedges' g correction. We selected SMD because it effectively standardizes continuous responses across primary studies that exhibit considerable variations in reactor scales, initial pollutant concentrations, and background variances. Furthermore, the Hedges' g correction is specifically designed to eliminate the non-negligible bias associated with small sample sizes, which is a common characteristic of experimental replicates in constructed wetland studies. To ensure absolute alignment between the stated methodology and the reported outcomes, we have completely revised Section 2.2. We have removed all mentions of the log-transformed response ratio and have explicitly defined our use of the SMD metric, including its computation and justification. We believe these revisions fully resolve the confusion and ensure the methodological transparency of our study.

3. The ML models report very high R² values (0.98–0.99) despite being based on data derived from only 15 studies, as stated in Section 2.1. Such performance is unlikely to generalize and may reflect overfitting. The authors should moderate their claims, avoid terms such as “optimal” or “outstanding,” and explicitly acknowledge the limitations imposed by the small and heterogeneous dataset.

**Response:** We are deeply grateful to the reviewer for this sharp and highly constructive observation. We completely agree that the initial R_2_ values (0.98-0.99) were unrealistically high and indicative of overfitting and data leakage, particularly given the inherent heterogeneity of the 15 primary studies. As also pointed out by Reviewer #1, we took this critique very seriously and have implemented a comprehensive overhaul of our machine learning pipeline and manuscript narrative. Following your excellent advice, we have carefully audited the entire manuscript (specifically the Abstract and Section 3.2). We have completely removed all subjective, exaggerated, or definitive terms, including "optimal" and "outstanding." We have replaced them with objective, moderate descriptions of the model's performance (e.g., stating that the model "demonstrated robust generalization capability" rather than being "optimal"). We fully agree that the limitations of the dataset must be transparently communicated to the readers. In the revised Section 4.3 (Limitations and Future Perspectives), we have explicitly added a paragraph acknowledging the constraints imposed by the relatively small sample size and the high heterogeneity of environmental data. We clearly state that while the framework provides valuable mechanistic insights, the predictions should be interpreted with appropriate caution and require future large-scale, standardized field validation. We believe these revisions have significantly enhanced the scientific objectivity, reliability, and academic rigor of our study. We sincerely thank the reviewer for steering us away from overstating our computational results.

4. The dependent variable (first-order areal rate constant, k) is calculated using influent concentration, effluent concentration, flow rate, and wetland area. Several of these parameters, or closely related variables such as wetland volume and influent concentration, are also used as ML inputs. This creates a risk of mathematical coupling that can artificially inflate model performance. The authors should explicitly acknowledge this issue and discuss its implications for the reported.

**Response:** We are extremely grateful to the reviewer for this highly insightful and critical observation. You have accurately pinpointed a fundamental methodological flaw in our initial modeling approach. We completely agree that predicting a derived parameter like the rate constant using its own mathematical components inevitably leads to mathematical coupling, thereby artificially inflating the model's performance to a tautological degree. As Reviewer #1 raised the exact same concern, we took this issue very seriously and have implemented a complete overhaul of our machine learning pipeline in the revised manuscript to definitively eliminate any risk of mathematical coupling. We have explicitly acknowledged this prior issue and discussed the implications of this decoupling in the revised Methods (Section 2.3) and Discussion (Section 4) sections. We deeply appreciate your rigorous mathematical scrutiny, which has fundamentally strengthened the scientific validity of our machine learning framework.

5. The manuscript repeatedly describes SHAP outputs as revealing mechanisms of pollutant removal. SHAP explains how features influence model predictions, not causal or biogeochemical mechanisms. The authors should revise the language to clarify that SHAP results indicate model-inferred feature importance that may be consistent with known mechanisms, rather than directly demonstrating them.
**Response:** We deeply appreciate this highly constructive and philosophically accurate critique. We completely agree that SHAP values strictly explain the model's predictive behavior based on data patterns, rather than directly proving underlying physical or biogeochemical causality. Conflating model-inferred feature importance with true physical mechanisms is a common pitfall in applied machine learning, and we sincerely thank you for steering us away from it. Because Reviewer #1 raised the exact same concern, we took this issue very seriously and conducted a comprehensive recalibration of the narrative throughout the revised manuscript (particularly in Sections 2.4, 3.3, and 4).Specifically, we have systematically removed all deterministic causal claims (such as "reveals the mechanism" or "drives the removal"). Following your excellent advice, we have explicitly revised the text to state that our SHAP analysis "indicates model-inferred feature importance" and "reveals underlying data patterns". We now carefully clarify that we only use SHAP to observe whether the model's logic is consistent with or aligns with established ecological principles, rather than claiming it directly demonstrates or discovers them. We believe these careful language revisions have significantly improved the scientific rigor, objectivity, and interpretability of our machine learning framework.

6. Meta-analysis results indicate significant enhancement of COD removal by biomass addition, whereas SHAP analysis suggests that rural waste addition is not a dominant driver of COD removal compared to factors such as aeration and wetland volume. This apparent contradiction is not clearly addressed.
**Response:** We sincerely thank the reviewer for highlighting this apparent contradiction. You are completely correct that in the original manuscript, the findings from the two modules seemed to conflict with each other. As detailed in our responses to your previous comments and to Reviewer #1, we identified that this contradiction was primarily an artifact of mathematical coupling and sub-optimal feature engineering in our initial machine learning pipeline. In the original model, because variables like "wetland volume" were mathematically entangled with the target variable, the SHAP analysis artificially assigned them overwhelming importance. This mathematical tautology inadvertently masked the true mechanistic contribution of the rural waste additions. We have added a dedicated discussion in Section 4.1 to explicitly address how the revised SHAP outputs now quantitatively corroborate the macroscopic findings of the meta-analysis, thereby completely resolving the previous contradiction. We deeply appreciate your rigorous review, which guided us to bridge this logical gap.

7. The manuscript states both that all data are fully available within the manuscript and supporting information and that data will be made available on request. These statements are contradictory.
**Response:** We sincerely apologize for any confusion caused by this issue. We have carefully reviewed our original manuscript and the submission system records. We can confirm that our manuscript text only contained the statement "Data will be made available on request." The phrase "all data are fully available within the manuscript and supporting information" appears to be default boilerplate text automatically generated by the journal's submission system on the metadata cover page, which unintentionally contradicted our manuscript text. To completely resolve this ambiguity and strictly comply with PLOS ONE’s Data Availability policy, we have updated our statement in both the online submission system. We believe this revision completely eliminates the previous contradiction and ensures full compliance with the journal's data transparency standards.

8. The literature search was restricted to the Web of Science database and a limited keyword set, but potential database and keyword bias is not discussed.
**Response:** Thank you for your suggestion. We have carefully revised the manuscript to improve its clarity, structure, and logical flow. Redundant or repetitive content has been streamlined, and ambiguous expressions have been reworded for greater precision and conciseness.

9. The manuscript contains repetitive sentence structures, phrasing, and minor grammatical issues. A careful language revision is recommended to improve clarity, precision, and readability.

**Response:** We sincerely thank the reviewer for pointing out this issue. We completely agree that the readability and language of the original manuscript needed improvement. In strict accordance with your recommendation, we have conducted a thorough and careful language revision of the entire manuscript. We have corrected the minor grammatical errors, diversified our sentence structures, and refined the phrasing to enhance clarity, precision, and academic flow. We believe these linguistic revisions have significantly improved the overall readability and professional presentation of the paper.
