# Supplementary material for: Pollution removal efficiency enhancement by agricultural biomass additions in constructed wetlands: A framework integrating meta-analysis with explainable machine learning
Source: PLoS One. 2026 Jul 7;21(7):e0353064. doi: 10.1371/journal.pone.0353064 (PMC13340836; doi:10.1371/journal.pone.0353064)
Supplement: Attachment — Submitted filename: Response to Reviews_2.docx [file pone.0353064.s006.docx]

May 7^th^, 2026

Dear Editor and Reviewers:

Enclosed please find the revised manuscript (PONE-D-26-02227), “Pollution Removal Efficiency Enhancement by Agricultural Biomass Additions in Constructed Wetlands: A Framework Integrating Meta-Analysis with Explainable Machine Learning” by Wenjie Li, Jian Wang, Xinya Liu, Yulin Zhuang, Hongda Fang, Jinliang Huang. We appreciate the time and efforts you and the reviewers have dedicated to providing valuable feedback on our manuscript. We have ensured that each comment has been carefully addressed and the revised manuscript is now clearer and stronger. We are now submitting it for further consideration for publication in your esteemed journal. Please note that the line number in each response corresponds to the line number in the revised manuscript with marked changes.

**Reviewer #1:**

**General comments:**

1. The graphical abstract still displays R^2^=0.99 for TN and R^2^=0.98 for COD, while the revised manuscript text now reports R^2^=0.83 and R²=0.76 respectively. The graphical abstract must be regenerated from the revised computational outputs.

**Response:** We sincerely apologize for this oversight. You are absolutely correct. While we thoroughly updated the manuscript text and the main figures to reflect the new cross-validated results, we neglected to synchronize the Graphical Abstract. We have now regenerated the Graphical Abstract using the revised computational outputs. The updated image accurately displays the corrected performance metrics (R^2^=0.83 for the XGBoost model simulating TN removal, and R^2^=0.76 for the Random Forest model simulating COD removal), fully aligning with the main text. We deeply appreciate your meticulous attention to detail, which has helped us ensure complete consistency throughout all components of our submission.

2. The authors claim in their rebuttal to have implemented “nested cross-validation” and “study-level grouped cross-validation,” yet Section 2.3 of the revised manuscript still describes a standard 70/30 train-validation split with 5-fold CV and five repeats. There is no description of inner/outer loops, no mention of study-level grouping, and no reference to GroupKFold or equivalent implementation. The methods text directly contradicts the rebuttal claims. The authors should consult literature for proper implementation and reporting of grouped CV strategies for clustered environmental data.

**Response:** We sincerely apologize for this glaring discrepancy and deeply appreciate your meticulous review. You are absolutely right to call this out. While we had indeed completely overhauled our code to implement the nested and study-level grouped cross-validation prior to our previous submission, we unfortunately failed to update the corresponding methodology text in Section 2.3. It was a pure drafting oversight on our part, which regrettably left the outdated "70/30 split" text in the manuscript, directly contradicting our rebuttal claims. We have now completely rewritten Section 2.3 (Implementation of ML models) to accurately and transparently describe the computational pipeline that was actually executed. We have checked the manuscript to ensure it is now perfectly aligned with our actual methodology and the provided code repository (https://github.com/leeeee-max/Plos-One.git). Thank you for holding us to the highest standards of reporting accuracy; your sharp attention to detail has been invaluable in finalizing this manus

3. The NLR formula correction and the shift from the rate constant k to removal efficiency as the prediction target are presented in the rebuttal as typographical fixes that did not affect the actual computational pipeline. This is a substantial claim; the authors are asserting that the original equations were wrong in the text but correct in their code, and that downstream results remain valid. Without providing verifiable evidence (e.g., code commits, audit logs, or at minimum the corrected code in the GitHub repository), this claim cannot be independently assessed. The authors should ensure the public repository reflects the described pipeline in its entirety.

**Response:** We sincerely thank the reviewer for this sharp, highly justified, and necessary critique. You are absolutely correct to question our previous characterization of these critical changes as mere "typographical fixes." We deeply apologize for our poor choice of words in the previous rebuttal, which inadvertently downplayed the reality and extent of the revisions we performed.

To be completely transparent regarding our workflow and correct the record:

During our original manuscript submission, we did not provide the underlying code. At that time, the original manuscript indeed contained the flawed NLR formula and erroneously targeted the first-order rate constant k.

However, during the first round of revisions, directly prompted by the rigorous and constructive feedback from the reviewers. we conducted a comprehensive overhaul of our actual computational pipeline. We did not simply fix typos; we explicitly rewrote the programming scripts to correct the NLR formula (removing the redundant HRT term entirely) and fundamentally shifted the machine learning prediction target exclusively to absolute removal percentages. Therefore, the updated model outputs reported in our revised manuscript, specifically the R^2^=0.76 for COD and R^2^=0.82 for TN are the direct computational results of this newly overhauled and executed code, not the original pipeline. We apologize again for the confusion caused by our previous rebuttal wording.

4. The citation base for the ML methodology is narrow and somewhat dated. The authors should engage with more recent literature on ML interpretability in water treatment contexts, automated ML with SHAP-based interpretation for pollutant removal, and ML combined with meta-heuristic optimization for wastewater systems. Additionally, the meta-analysis methodology would benefit from citing established references on effect size computation and Hedges’ g correction beyond the general introductory references currently provided. The SHAP interpretation sections should also draw on the broader interpretable ML literature to properly frame the distinction between model explanation and causal inference. References the authors may wish to consult: Introduction to Meta-Analysis. Wiley, (2009).

Roberts, D.R. et al. Cross-validation strategies for data with temporal, spatial, hierarchical, or phylogenetic structure. Ecography, 40(8), 913-929, (2017); Interpretable Machine Learning: A Guide for Making Black Box Models Explainable. 2nd ed., (2022); A review on applications of biochar in constructed wetlands. Journal of Cleaner Production, 394, 136257, (2023); Machine Learning for Electrochemical Advanced Oxidation in Water Treatment: Descriptors, Interpretability, and Control. ACS ES&T Water, (2026); Automated machine learning and SHAP-based interpretation of PFOA removal via electrochemical oxidation. Desalination and Water Treatment, 101598, (2025).

**Response:** Thank you for your suggestion. We are deeply grateful to the reviewer for this sharp and highly critical observation. We fully agree that the initial R^2^ values of 0.98 were a clear red flag. Upon rigorous re-evaluation, we identified that this artificially inflated performance was caused by a combination of mathematical coupling in the feature space and data leakage stemming from standard random data splitting across clustered literature datasets. To address this fundamental methodological flaw, we have completely overhauled our machine learning pipeline and implemented a rigorous, leakage-free validation strategy in the revised manuscript. We have abandoned the previous ambiguous train-validation split. Instead, we established a strict nested cross-validation framework. Hyperparameter tuning is now strictly confined to the inner cross-validation loop. The optimized model is then evaluated exclusively on the completely untouched outer loop hold-out sets. This guarantees an absolute separation between model tuning and out-of-sample validation. To prevent data leakage where observations from the same primary study appear in both the training and testing sets, we replaced simple random splitting with Study-Level Grouped Cross-Validation. The models are now trained on a subset of studies and tested on entirely unseen studies, ensuring that the validation metrics reflect true external generalization. We have updated the Results section (Section 3.2), the Abstract, and the relevant Figures to reflect these new metrics. Furthermore, we have completely removed exaggerated claims such as "optimal" or "outstanding" from the text. We sincerely thank the reviewer for guiding us to significantly improve the rigor of our computational framework.

We sincerely appreciate this highly constructive feedback and thank you for providing such an excellent and highly relevant list of recommended literature. We fully agree that our original citation base was too narrow and did not adequately capture the most recent methodological advancements or the foundational statistical texts required to rigorously frame our study. Following your excellent suggestions, we have comprehensively updated our bibliography and enriched the corresponding discussions throughout the revised manuscript. Specifically, we have integrated your recommended references (alongside other recent studies) into the following sections:

Meta-Analysis Methodology (Section 2.2): We have cited the foundational text (Introduction to Meta-Analysis, Wiley, 2009) to provide a rigorous and established theoretical basis for our effect size computation and the specific application of the Hedges' g correction for small sample sizes.

Cross-Validation Strategy (Section 2.3): To properly ground our newly implemented study-level grouped cross-validation strategy, we have explicitly cited Roberts et al. (2017). This accurately justifies our approach to handling the hierarchical and clustered structure of the environmental data extracted from the primary studies.

Advanced ML in Water Treatment (Sections 1 & 4): We have engaged with the recent 2025 and 2026 literature you recommended (concerning automated ML, SHAP interpretation, and advanced oxidation in water treatment). Incorporating these state-of-the-art studies has significantly broadened our framing and demonstrated the wider applicability of explainable ML frameworks in wastewater systems.

SHAP Interpretation vs. Causal Inference (Sections 2.4 & 4): Drawing upon the Interpretable Machine Learning (2022) text, we have fundamentally refined our language regarding the SHAP analysis. We now use this literature to explicitly frame our SHAP results strictly as "model explanation" and "model-inferred feature importance," clearly distinguishing it from true physical or biogeochemical causal inference.

We are extremely grateful for these reading recommendations. They have significantly elevated the academic rigor, contextual depth, and current relevance of our methodological framework.

5. The response to Reviewer #2’s Comment 8 regarding database and keyword bias is a complete non-answer. The reviewer asked specifically about the limitation of restricting the literature search to Web of Science with a narrow keyword set. The authors responded with a generic statement about improving clarity and streamlining content, entirely dodging the substantive concern. While the revised Limitations section does now briefly mention the WoS restriction, the evasiveness of the rebuttal response is concerning and suggests the authors may not have fully engaged with the underlying methodological critique.

**Response:** We sincerely thank the reviewer for pointing out this glaring omission. You are absolutely correct that our previous response to Reviewer #2’s Comment 8 was a complete non-answer. We offer our deepest apologies for this. It was a highly regrettable clerical and drafting error during the compilation of the previous rebuttal: we accidentally pasted a generic formatting response (intended for a language/structure comment) into that section, completely overwriting our substantive explanation. We fully understand why this appeared evasive, and we assure you it was not our intention to dodge this critical methodological critique.

Our initial decision to restrict the search exclusively to the Web of Science (WoS) Core Collection was driven by the strict data quality requirements of our explainable machine learning framework. We required studies that reported highly detailed and standardized operational matrices (e.g., precise wetland volumes, HRT, and biomass physicochemical properties). However, we fully agree that this single-database approach introduces an inevitable database bias. It likely excluded highly relevant and high-quality empirical studies indexed in Scopus, Engineering Village, or regional databases (such as CNKI), potentially skewing the findings toward specific geographic regions or dominant research groups. To demonstrate our full engagement with this crucial methodological limitation, we have significantly expanded the "Limitations and future perspectives" section (Section 4.3) in the revised manuscript. The revised text now deeply reflects on how these database and keyword biases may impact the generalizability of our findings. We are extremely grateful for your rigorous oversight in ensuring this limitation is properly documented.
