# Supplementary material for: Pollution removal efficiency enhancement by agricultural biomass additions in constructed wetlands: A framework integrating meta-analysis with explainable machine learning
Source: PLoS One. 2026 Jul 7;21(7):e0353064. doi: 10.1371/journal.pone.0353064 (PMC13340836; doi:10.1371/journal.pone.0353064)
Supplement: Peer review history

# Peer review: Decision Letter 0

**DOI:** 10.1371/journal.pone.0353064.r001

**Authors:** Sovik Das

15 Feb 2026

PONE-D-26-02227

Pollution Removal Efficiency Enhancement by Agricultural Biomass Additions in Constructed Wetlands: A Framework Integrating Meta-Analysis with Explainable Machine Learning

PLOS One

Dear Dr. Huang,

Thank you for submitting your manuscript to PLOS ONE. After careful consideration, we feel that it has merit but does not fully meet PLOS ONE’s publication criteria as it currently stands. Therefore, we invite you to submit a revised version of the manuscript that addresses the points raised during the review process.

Please submit your revised manuscript by Apr 01 2026 11:59PM. If you will need more time than this to complete your revisions, please reply to this message or contact the journal office at plosone@plos.org. When you're ready to submit your revision, log on to https://www.editorialmanager.com/pone/ and select the 'Submissions Needing Revision' folder to locate your manuscript file.

Please include the following items when submitting your revised manuscript:

- A letter that responds to each point raised by the academic editor and reviewer(s). You should upload this letter as a separate file labeled 'Response to Reviewers'.
- A marked-up copy of your manuscript that highlights changes made to the original version. You should upload this as a separate file labeled 'Revised Manuscript with Track Changes'.
- An unmarked version of your revised paper without tracked changes. You should upload this as a separate file labeled 'Manuscript'.

If you would like to make changes to your financial disclosure, please include your updated statement in your cover letter. Guidelines for resubmitting your figure files are available below the reviewer comments at the end of this letter.

If applicable, we recommend that you deposit your laboratory protocols in protocols.io to enhance the reproducibility of your results. Protocols.io assigns your protocol its own identifier (DOI) so that it can be cited independently in the future. For instructions see: https://journals.plos.org/plosone/s/submission-guidelines#loc-laboratory-protocols. Additionally, PLOS ONE offers an option for publishing peer-reviewed Lab Protocol articles, which describe protocols hosted on protocols.io. Read more information on sharing protocols at https://plos.org/protocols?utm_medium=editorial-email&utm_source=authorletters&utm_campaign=protocols.

We look forward to receiving your revised manuscript.

Kind regards,

Sovik Das

Academic Editor

PLOS One

Journal Requirements:

When submitting your revision, we need you to address these additional requirements.

1. Please ensure that your manuscript meets PLOS ONE's style requirements, including those for file naming. The PLOS ONE style templates can be found at

https://journals.plos.org/plosone/s/file?id=wjVg/PLOSOne_formatting_sample_main_body.pdf and

https://journals.plos.org/plosone/s/file?id=ba62/PLOSOne_formatting_sample_title_authors_affiliations.pdf

2. Please note that PLOS One has specific guidelines on code sharing for submissions in which author-generated code underpins the findings in the manuscript. In these cases, we expect all author-generated code to be made available without restrictions upon publication of the work. Please review our guidelines at https://journals.plos.org/plosone/s/materials-and-software-sharing#loc-sharing-code and ensure that your code is shared in a way that follows best practice and facilitates reproducibility and reuse.

3. Thank you for stating the following in the Acknowledgments Section of your manuscript:

“This research was supported by the Natation Natural Science Foundation of China (Grant No.42376225) and Fujian Environmental Protection Science and Technology Plan Project(Grant 2022R007).”

We note that you have provided funding information that is not currently declared in your Funding Statement. However, funding information should not appear in the Acknowledgments section or other areas of your manuscript. We will only publish funding information present in the Funding Statement section of the online submission form.

Please remove any funding-related text from the manuscript and let us know how you would like to update your Funding Statement. Currently, your Funding Statement reads as follows:

“The author(s) received no specific funding for this work.”

Please include your amended statements within your cover letter; we will change the online submission form on your behalf.

4. We note that the grant information you provided in the ‘Funding Information’ and ‘Financial Disclosure’ sections do not match.

When you resubmit, please ensure that you provide the correct grant numbers for the awards you received for your study in the ‘Funding Information’ section.

5. Thank you for stating the following financial disclosure:

“This research was supported by the Natation Natural Science Foundation of China (Grant No.42376225) and Fujian Environmental Protection Science and Technology Plan Project(Grant 2022R007).”

At this time, please address the following queries:

a) Please clarify the sources of funding (financial or material support) for your study. List the grants or organizations that supported your study, including funding received from your institution.

b) State what role the funders took in the study. If the funders had no role in your study, please state: “The funders had no role in study design, data collection and analysis, decision to publish, or preparation of the manuscript.”

c) If any authors received a salary from any of your funders, please state which authors and which funders.

d) If you did not receive any funding for this study, please state: “The authors received no specific funding for this work.”

Please include your amended statements within your cover letter; we will change the online submission form on your behalf.

6. In the online submission form you indicate that your data is not available for proprietary reasons and have provided a contact point for accessing this data. Please note that your current contact point is a co-author on this manuscript. According to our Data Policy, the contact point must not be an author on the manuscript and must be an institutional contact, ideally not an individual. Please revise your data statement to a non-author institutional point of contact, such as a data access or ethics committee, and send this to us via return email. Please also include contact information for the third party organization, and please include the full citation of where the data can be found.

7. Please include your full ethics statement in the ‘Methods’ section of your manuscript file. In your statement, please include the full name of the IRB or ethics committee who approved or waived your study, as well as whether or not you obtained informed written or verbal consent. If consent was waived for your study, please include this information in your statement as well.

8. Please include a separate caption for each figure in your manuscript.

9. Please include captions for your Supporting Information files at the end of your manuscript, and update any in-text citations to match accordingly. Please see our Supporting Information guidelines for more information: http://journals.plos.org/plosone/s/supporting-information.

10. If the reviewer comments include a recommendation to cite specific previously published works, please review and evaluate these publications to determine whether they are relevant and should be cited. There is no requirement to cite these works unless the editor has indicated otherwise.

[Note: HTML markup is below. Please do not edit.]

Reviewers' comments:

Reviewer's Responses to Questions

**Comments to the Author**

1. Is the manuscript technically sound, and do the data support the conclusions?

The manuscript must describe a technically sound piece of scientific research with data that supports the conclusions. Experiments must have been conducted rigorously, with appropriate controls, replication, and sample sizes. The conclusions must be drawn appropriately based on the data presented.

Reviewer #1: No

Reviewer #2: Yes

**********

2. Has the statistical analysis been performed appropriately and rigorously?

Reviewer #1: No

Reviewer #2: No

**********

3. Have the authors made all data underlying the findings in their manuscript fully available?

The PLOS Data policy requires authors to make all data underlying the findings described in their manuscript fully available without restriction, with rare exception (please refer to the Data Availability Statement in the manuscript PDF file). The data should be provided as part of the manuscript or its supporting information, or deposited to a public repository. For example, in addition to summary statistics, the data points behind means, medians and variance measures should be available. If there are restrictions on publicly sharing data—e.g. participant privacy or use of data from a third party—those must be specified.

Reviewer #1: No

Reviewer #2: Yes

**********

4. Is the manuscript presented in an intelligible fashion and written in standard English?

PLOS ONE does not copyedit accepted manuscripts, so the language in submitted articles must be clear, correct, and unambiguous. Any typographical or grammatical errors should be corrected at revision, so please note any specific errors here.

Reviewer #1: No

Reviewer #2: Yes

**********

5. Review Comments to the Author

Please use the space provided to explain your answers to the questions above. You may also include additional comments for the author, including concerns about dual publication, research ethics, or publication ethics. (Please upload your review as an attachment if it exceeds 20,000 characters)

Reviewer #1: Your manuscript tackles a genuinely worthwhile idea regarding using agricultural biomass in constructed wetlands to improve performance under low C/N conditions, and the “meta-analysis + explainable ML” framing could have been a strong contribution. Unfortunately, the paper in its current form reads like two separate studies stapled together, with major inconsistencies in definitions, equations, and what exactly is being predicted.

The first problem is internal inconsistency in the meta-analysis. The manuscript states that a log-transformed response ratio is used to quantify effects, yet the headline results are reported as “SMD” values (e.g., SMD = 2.50, SMD = 1.20) and interpreted as if SMD were the core effect metric. If you used standardized mean difference, you must define it, justify it for this context, and show the exact computation. If you used a response ratio, then the reporting and interpretation must match that choice. Right now the paper communicates one metric in the methods and a different metric in the results, and the reader is left unable to verify what was actually done.

Closely related, key equations appear incorrect or at least unjustified in their current form. Your “NLR” definition includes both wetland volume and HRT in the denominator, even though HRT is already volume-dependent in the usual formulation. As written, the units and physical meaning become questionable, and that means the downstream effect sizes are being computed from a possibly misdefined performance variable. If you want to normalize removal by reactor volume, there is a straightforward way to do it; what you have currently needs to be re-derived carefully, with dimensional checking and a clear statement of why this form is appropriate.

The literature search and screening process also needs much tighter reporting. The Boolean logic in the search string is written in a way that can easily retrieve papers that match only parts of the intended condition (because of operator precedence). A reader should not have to “trust” that the right set of studies was retrieved; the search expression must be unambiguous, reproducible, and correctly bracketed. You also need to be explicit about what you mean by “low C/N” (thresholds, measurement basis, and how you treated studies that report ranges or variable influent conditions), because your entire premise hinges on that classification.

On the machine-learning side, the paper currently oversells performance in a way that raises immediate validity concerns. Reporting R2 values near 0.98–0.99 in an environmental dataset assembled from heterogeneous studies is a red flag unless you demonstrate, very explicitly, that there is no leakage and that the validation strategy reflects genuine out-of-sample generalization. The manuscript states both a 70/30 train–validation split and repeated 5-fold cross-validation with tuning, but it is not clear how those were combined (and whether tuning was nested properly). Without a clean separation between training, tuning, and final hold-out evaluation, the resulting metrics can look excellent while being inflated.

Even more fundamentally, the target variable and the narrative do not line up. In some places you describe predicting COD/TN removal; elsewhere you describe predicting first-order areal rate coefficients (k). Those are not interchangeable, and the features you list include variables that can be mathematically entangled with the target (for example, quantities derived from influent/effluent concentrations, geometry, flow, or retention time depending on how k is computed). If the model is effectively learning a rearranged version of the same equation used to compute the target, very high R² is not a scientific achievement; it is a tautology. You need to clearly define the prediction target, show exactly how it is computed, list which inputs are used, and explain why the prediction is not simply reconstructing an identity.

The SHAP section suffers from the same problem: interpretability is only meaningful if the prediction task is well-posed and the features are not proxies for the target by construction. Right now the SHAP narrative risks becoming a sophisticated-looking commentary on variable correlations rather than a defensible explanation of causal or mechanistic drivers. If you want readers to trust the interpretability claims, you need to show robustness checks (for example, sensitivity to feature sets, study-level blocking, and performance under strict external validation), and you need to be careful about the language: SHAP explains the model, not necessarily the system. You may check the following: (2024). The crucial factor for microplastics removal in large-scale subsurface-flow constructed wetlands. Journal of Hazardous Materials, 480, 136023; Automated machine learning and SHAP-based interpretation of PFOA removal via electrochemical oxidation. Desalination and Water Treatment, 325, 101598; Hybrid machine learning model with SHAP interpretability for optimization of targeted nitrogen and phosphorus removal from bioretention systems. Journal of Water Process Engineering, 77, 108417.

Finally, the manuscript’s central promise (integrating meta-analysis with explainable ML) needs to be made real rather than rhetorical. At the moment, the meta-analysis produces claims about “best biomass types,” while the ML produces claims about “key drivers,” but the bridge between them is weak. If the ML dataset is drawn from only a subset of the meta-analysis studies, explain why. If biomass type/pretreatment is a major conclusion in meta-analysis, it should be treated coherently in the ML feature design and interpretation. As written, the paper asks the reader to accept a unified framework but provides two partially inconsistent pipelines.

Reviewer #2: 1. The manuscript uses the terms datasets, studies, and observations inconsistently. The Abstract reports 272 and 1,283 datasets for meta-analysis and machine learning, whereas Section 2.1 states that 70 studies were used for meta-analysis and 15 studies for ML. The relationship between these numbers is not defined. The authors should clearly explain what constitutes a dataset, how many observations were extracted per study, and ensure consistent terminology throughout the manuscript.

2. Section 2.2 states that a log-transformed response ratio was used to calculate effect sizes, but the Results section reports standardized mean difference (SMD) values. These are different statistical measures and cannot be used interchangeably. The authors must clarify which effect size metric was actually applied and revise either the Methods or Results so that the reported outcomes align with the stated methodology.

3. The ML models report very high R² values (0.98–0.99) despite being based on data derived from only 15 studies, as stated in Section 2.1. Such performance is unlikely to generalize and may reflect overfitting. The authors should moderate their claims, avoid terms such as “optimal” or “outstanding,” and explicitly acknowledge the limitations imposed by the small and heterogeneous dataset.

4. The dependent variable (first-order areal rate constant, k) is calculated using influent concentration, effluent concentration, flow rate, and wetland area. Several of these parameters, or closely related variables such as wetland volume and influent concentration, are also used as ML inputs. This creates a risk of mathematical coupling that can artificially inflate model performance. The authors should explicitly acknowledge this issue and discuss its implications for the reported

5. The manuscript repeatedly describes SHAP outputs as revealing mechanisms of pollutant removal. SHAP explains how features influence model predictions, not causal or biogeochemical mechanisms. The authors should revise the language to clarify that SHAP results indicate model-inferred feature importance that may be consistent with known mechanisms, rather than directly demonstrating them.

6. Meta-analysis results indicate significant enhancement of COD removal by biomass addition, whereas SHAP analysis suggests that rural waste addition is not a dominant driver of COD removal compared to factors such as aeration and wetland volume. This apparent contradiction is not clearly addressed.

7. The manuscript states both that all data are fully available within the manuscript and supporting information and that data will be made available on request. These statements are contradictory.

8. The literature search was restricted to the Web of Science database and a limited keyword set, but potential database and keyword bias is not discussed.

9. The manuscript contains repetitive sentence structures, phrasing, and minor grammatical issues. A careful language revision is recommended to improve clarity, precision, and readability.

**********

6. PLOS authors have the option to publish the peer review history of their article (what does this mean?). If published, this will include your full peer review and any attached files.

If you choose “no”, your identity will remain anonymous but your review may still be made public.

**Do you want your identity to be public for this peer review?**  For information about this choice, including consent withdrawal, please see our Privacy Policy.

Reviewer #1: No

Reviewer #2: No

**********

[NOTE: If reviewer comments were submitted as an attachment file, they will be attached to this email and accessible via the submission site. Please log into your account, locate the manuscript record, and check for the action link "View Attachments". If this link does not appear, there are no attachment files.]

To ensure your figures meet our technical requirements, please review our figure guidelines: https://journals.plos.org/plosone/s/figures

You may also use PLOS’s free figure tool, NAAS, to help you prepare publication quality figures: https://journals.plos.org/plosone/s/figures#loc-tools-for-figure-preparation.

NAAS will assess whether your figures meet our technical requirements by comparing each figure against our figure specifications.

# Author comment: Author response to Decision Letter 1

**DOI:** 10.1371/journal.pone.0353064.r002

2 Apr 2026

March 27th, 2025

Dear Editor and Reviewers:

Enclosed please find the revised manuscript (PONE-D-26-02227), “Pollution Removal Efficiency Enhancement by Agricultural Biomass Additions in Constructed Wetlands: A Framework Integrating Meta-Analysis with Explainable Machine Learning” by Wenjie Li, Jian Wang, Xinya Liu, Yulin Zhuang, Hongda Fang, Jinliang Huang. We appreciate the time and efforts you and the reviewers have dedicated to providing valuable feedback on our manuscript. We have ensured that each comment has been carefully addressed and the revised manuscript is now clearer and stronger. We are now submitting it for further consideration for publication in your esteemed journal. Please note that the line number in each response corresponds to the line number in the revised manuscript with marked changes.

Updated Funding Statement:

This research was supported by the Natation Natural Science Foundation of China (Grant No.42376225) and Fujian Environmental Protection Science and Technology Plan Project(Grant 2022R007). The funders had no role in study design, data collection and analysis, decision to publish, or preparation of the manuscript.

Data Availability:

All data and author-generated code supporting the findings of this study are publicly available on GitHub at: https://github.com/leeeee-max/Plos-One.git. There are no restrictions on access.

Reviewer #1:

General comments:

Your manuscript tackles a genuinely worthwhile idea regarding using agricultural biomass in constructed wetlands to improve performance under low C/N conditions, and the “meta-analysis+explainable ML” framing could have been a strong contribution. Unfortunately, the paper in its current form reads like two separate studies stapled together, with major inconsistencies in definitions, equations, and what exactly is being predicted.

Response: We sincerely appreciate your positive comments. We have thoroughly revised the manuscript to improve its clarity, conciseness, and overall academic writing quality. We fully agree with your assessment and have implemented a comprehensive restructuring of the manuscript to explicitly demonstrate that this is a single, organically integrated framework designed to comprehensively answer our core scientific question: How can agricultural biomass addition optimize pollution removal in constructed wetlands under low C/N conditions? To clarify the indispensable connection between the two parts, we have revised the manuscript and structured our integration logic.

1. There is a direct contradiction between the methods section, which claims to use a log-transformed response ratio, and the results section, which reports Standardized Mean Difference (SMD). The metric must be uniform, clearly defined, and justified.

Response: Thank you for your suggestion. To clarify, the actual statistical metric calculated and utilized throughout our analysis was the Standardized Mean Difference (SMD), specifically applying the Hedges' g correction. The mention of the "log-transformed response ratio" in the original Methods section was an oversight during the manuscript drafting process and did not reflect the actual data pipeline we executed. We have now comprehensively revised the manuscript to ensure absolute uniformity. All references to the response ratio have been removed, and we have explicitly defined and justified our use of SMD in the revised Section 2.2 (Meta-Analysis). We have provided the exact computation formulas for the SMD and its variance estimation to ensure full transparency and reproducibility. We deeply appreciate the your meticulous reading, which has significantly tightened the methodological rigor of our paper.

2. The formula used for Nitrogen Loading Rate (NLR) is mathematically and physically flawed. Including both wetland volume and Hydraulic Retention Time (HRT) in the denominator causes redundant normalization (since HRT is already volume-dependent), compromising the validity of the downstream effect sizes.

Response: Thank you for your constructive suggestion. We have corrected the formula in the revised manuscript (Section 2.2). More importantly, regarding your highly valid concern about the validity of the downstream effect sizes and our machine learning models, we would like to explicitly clarify that this flawed equation was strictly a typographical and drafting error in the manuscript text, rather than a systematic flaw in our analytical pipeline. During our actual data extraction and computational processing, the mass removal rates were correctly computed based on the influent flow rate ($Q$) and the actual wetland volume, strictly avoiding the redundant incorporation of HRT. We have thoroughly re-audited our datasets and code scripts to ensure complete dimensional accuracy. We confirm that all computed effect sizes in the meta-analysis, as well as the input features for the machine learning models, were based on this correct physical logic. Therefore, the downstream effect sizes, along with the SHAP interpretations highlighting the importance of wetland volume, remain entirely robust and unaffected by this textual oversight. We deeply appreciate your rigorous mathematical checking, which has helped us eliminate this confusing typo and ensure the technical soundness of the paper.

3. The methodology for study selection lacks transparency and reproducibility. Specifically, the Boolean search string has operator precedence issues, and the threshold/definition for "low C/N" (a foundational premise of the study) is not explicitly defined.

Response: We sincerely appreciate the reviewer's meticulous evaluation of our methodology. We have completely addressed both issues in the revised manuscript. Following your highly constructive advice, we have now explicitly defined this threshold and its biogeochemical justification in Section 2.1. Specifically, we have added the statement to the revised manuscript. We believe these revisions have fundamentally improved the transparency, reproducibility, and scientific rigor of our study selection process. Thank you again for pointing out these methodological gaps.

4. Reporting unusually high R2 values (0.98–0.99) on heterogeneous environmental data strongly suggests data leakage or overfitting. The manuscript fails to clearly separate training, hyperparameter tuning, and out-of-sample validation (e.g., nested cross-validation).

Response: Thank you for your suggestion. We are deeply grateful to the reviewer for this sharp and highly critical observation. We fully agree that the initial R2 values of 0.98 were a clear red flag. Upon rigorous re-evaluation, we identified that this artificially inflated performance was caused by a combination of mathematical coupling in the feature space and data leakage stemming from standard random data splitting across clustered literature datasets. To address this fundamental methodological flaw, we have completely overhauled our machine learning pipeline and implemented a rigorous, leakage-free validation strategy in the revised manuscript. We have abandoned the previous ambiguous train-validation split. Instead, we established a strict nested cross-validation framework. Hyperparameter tuning is now strictly confined to the inner cross-validation loop. The optimized model is then evaluated exclusively on the completely untouched outer loop hold-out sets. This guarantees an absolute separation between model tuning and out-of-sample validation. To prevent data leakage where observations from the same primary study appear in both the training and testing sets, we replaced simple random splitting with Study-Level Grouped Cross-Validation. The models are now trained on a subset of studies and tested on entirely unseen studies, ensuring that the validation metrics reflect true external generalization. We have updated the Results section (Section 3.2), the Abstract, and the relevant Figures to reflect these new metrics. Furthermore, we have completely removed exaggerated claims such as "optimal" or "outstanding" from the text. We sincerely thank the reviewer for guiding us to significantly improve the rigor of our computational framework.

5. The prediction target is inconsistently described (removal efficiency vs. first-order rate constant k). More critically, the input features include variables mathematically entangled with the target. This means the model might simply be learning a rearranged mathematical identity rather than discovering genuine predictive relationships (a tautology).

Response: We are deeply grateful to the reviewer for this highly professional and critical observation. To completely resolve this issue and ensure the scientific validity of our ML models, we have implemented a comprehensive overhaul of Section 2.3 and the corresponding Results section. We have removed all equations and mentions regarding the prediction of the first-order areal rate constant. In the revised manuscript, we explicitly clarify that the sole prediction targets for our machine learning models are the absolute TN and COD Removal Efficiencies (%). This strategic decoupling forces the XGBoost and Random Forest algorithms to genuinely learn the underlying non-linear biogeochemical relationships and treatment dynamics, rather than merely reverse-engineering an algebraic identity. Consequently, the SHAP analysis now provides defensible, mechanistic insights into how specific operational drivers causally influence the overall pollutant removal performance. We believe these critical modifications have fundamentally strengthened the robustness and interpretability of our machine learning framework.

6. The SHAP analysis currently reads as a causal or mechanistic explanation of the physical system, rather than what it actually is: an explanation of the model. Because the input features may be proxies for the target, the SHAP results lack validity without strict robustness checks and external validation.

Response: We deeply appreciate this profound methodological and philosophical critique. We fully agree with your assessment. It is a common pitfall in applied machine learning to conflate model-inferred feature importance with true physical causality. SHAP strictly explains how the model reaches its specific predictions based on the given data, and we agree that it should not be overstated as a direct proof of biogeochemical mechanisms. To rigorously address this crucial point, we have implemented a comprehensive revision in our manuscript (specifically in Sections 2.4, 3.3, and 4). We have meticulously revised the language throughout the manuscript to remove any deterministic causal claims. Phrases such as "reveals the mechanism" or "drives the removal" have been replaced. Instead, we now explicitly state that SHAP is utilized for revealing the underlying data pattern and model-inferred feature interpretation. We have clarified that while SHAP explains the model's behavior, we only use it to observe whether the model's logic aligns with established ecological principles, rather than claiming it discovers them. Regarding your highly valid concern that input features might simply be proxies for the target, this issue was fundamentally resolved by our structural revision (as detailed in our response to your previous comments regarding mathematical coupling). By shifting the prediction target exclusively to the absolute Removal Efficiency and eliminating all mathematically coupled variables, our input features are now strictly independent operational and environmental parameters. They are no longer mathematical proxies for the target, ensuring that the SHAP values reflect genuine operational influence rather than tautological relationships.

7. The manuscript promises a unified framework but delivers two disconnected pipelines. The key findings from the meta-analysis (e.g., optimal biomass types) are not coherently integrated into the feature engineering or the interpretations of the machine learning model.

Response: We are extremely grateful for this insightful critique. We fully agree that in the original manuscript, the narrative bridge between the meta-analysis findings and the machine learning (ML) feature engineering was not articulated clearly, giving the unintended impression of two disconnected pipelines. We have completely rewritten the transition between these two modules (Section 3.2 and Section 4.1) to explicitly demonstrate how they form a single, coherent, and sequential framework. You rightfully pointed out that if "biomass type" is the major conclusion of the meta-analysis, it should be reflected in the ML features. In the revised Section 3.2 (Feature Engineering), we now explicitly explain our feature selection rationale: rather than feeding categorical labels (e.g., "bamboo" or "lotus leaf") into the XGBoost model, which limits generalizability to unseen materials, we quantitatively translated the optimal traits identified in the meta-analysis into continuous operational features. Specifically, the distinct physicochemical properties of different biomass types were engineered into the features "biochar COD release" and "biochar TN release". This represents a direct, coherent data bridge: the meta-analysis identified which materials work best qualitatively, and the ML feature engineering captured why they work quantitatively. In the revised Discussion (Section 4.1), we have harmonized the interpretation. The SHAP analysis revealed that "TN release from the added carbon source" is a top-three influential feature. We now explicitly discuss how this ML finding perfectly corroborates the meta-analysis finding: the superiority of lotus leaf and bamboo biochar (found in the meta-analysis) is mathematically driven by their specific carbon/nitrogen release kinetics and highly porous structures (validated by the ML/SHAP analysis).

Reviewer #2:

1. The manuscript uses the terms datasets, studies, and observations inconsistently. The Abstract reports 272 and 1,283 datasets for meta-analysis and machine learning, whereas Section 2.1 states that 70 studies were used for meta-analysis and 15 studies for ML. The relationship between these numbers is not defined. The authors should clearly explain what constitutes a dataset, how many observations were extracted per study, and ensure consistent terminology throughout the manuscript.

Response: We sincerely thank the reviewer for pointing out this critical issue regarding terminology inconsistency, and we apologize for the confusion caused by our previous misuse of the word "datasets" to refer to individual "observations." We fully agree that precise data terminology is essential for the reproducibility and clarity of this framework. To address your concern, we have completely standardized our terminology throughout the revised manuscript (specifically in the Abstract and Section 2.1) by strictly defining a "study" as an independent, peer-reviewed publication retrieved from our literature search, an "observation" as a single experimental measurement extracted from a study, and a "dataset" as the entire compiled matrix of observations used to execute a specific analytical algorithm. Based on these strict definitions, we have clarified the relationships between the reported numbers: for the meta-analysis, we screened 70 studies to extract a total of 272 independent observations (averaging approximately 3.9 observations per study), which collectively constitute our "meta-analysis dataset." For the machine learning (ML) analysis, we selected a subset of 15 studies from this pool because they reported the highly detailed, high-dimensional operational parameters required by ML models. Consequently, we were able to extract a substantial 1,283 independent observations from just these 15 studies (averaging approximately 85.5 observations per study) to form our "ML dataset." Accordingly, we have corrected the Abstract to state "...using 272 and 1,283 independent observations extracted from 70 and 15 published studies for meta-analysis and machine learning, respectively," and we have explicitly added a paragraph in Section 2.1 defining these relationships and the average number of observations extracted per study. We believe these corrections completely remove any ambiguous use of the word "datasets," eliminating previous co

Attachment Submitted filename: Response to Reviews.docx

# Peer review: Decision Letter 1

**DOI:** 10.1371/journal.pone.0353064.r003

**Authors:** Sovik Das, Sovik Das

15 Apr 2026

PONE-D-26-02227R1

Pollution removal efficiency enhancement by agricultural biomass additions in constructed wetlands: A framework integrating meta-analysis with explainable machine learning

PLOS One

Dear Dr. Huang,

Thank you for submitting your manuscript to PLOS ONE. After careful consideration, we feel that it has merit but does not fully meet PLOS ONE’s publication criteria as it currently stands. Therefore, we invite you to submit a revised version of the manuscript that addresses the points raised during the review process.

Please submit your revised manuscript by May 30 2026 11:59PM. If you will need more time than this to complete your revisions, please reply to this message or contact the journal office at plosone@plos.org. When you're ready to submit your revision, log on to https://www.editorialmanager.com/pone/ and select the 'Submissions Needing Revision' folder to locate your manuscript file.

Please include the following items when submitting your revised manuscript:

- A letter that responds to each point raised by the academic editor and reviewer(s). You should upload this letter as a separate file labeled 'Response to Reviewers'.
- A marked-up copy of your manuscript that highlights changes made to the original version. You should upload this as a separate file labeled 'Revised Manuscript with Track Changes'.
- An unmarked version of your revised paper without tracked changes. You should upload this as a separate file labeled 'Manuscript'.

If you would like to make changes to your financial disclosure, please include your updated statement in your cover letter. Guidelines for resubmitting your figure files are available below the reviewer comments at the end of this letter.

If applicable, we recommend that you deposit your laboratory protocols in protocols.io to enhance the reproducibility of your results. Protocols.io assigns your protocol its own identifier (DOI) so that it can be cited independently in the future. For instructions see: https://journals.plos.org/plosone/s/submission-guidelines#loc-laboratory-protocols. Additionally, PLOS ONE offers an option for publishing peer-reviewed Lab Protocol articles, which describe protocols hosted on protocols.io. Read more information on sharing protocols at https://plos.org/protocols?utm_medium=editorial-email&utm_source=authorletters&utm_campaign=protocols.

As the corresponding author, your ORCID iD is verified in the submission system and will appear in the published article. PLOS supports the use of ORCID, and we encourage all coauthors to register for an ORCID iD and use it as well. Please encourage your coauthors to verify their ORCID iD within the submission system before final acceptance, as unverified ORCID iDs will not appear in the published article. *Only*  the individual author can complete the verification step; PLOS staff *cannot*  verify ORCID iDs on behalf of authors.

We look forward to receiving your revised manuscript.

Kind regards,

Sovik Das

Academic Editor

PLOS One

Journal Requirements:

If the reviewer comments include a recommendation to cite specific previously published works, please review and evaluate these publications to determine whether they are relevant and should be cited. There is no requirement to cite these works unless the editor has indicated otherwise.

[Note: HTML markup is below. Please do not edit.]

Reviewers' comments:

Reviewer's Responses to Questions

**Comments to the Author**

1. If the authors have adequately addressed your comments raised in a previous round of review and you feel that this manuscript is now acceptable for publication, you may indicate that here to bypass the “Comments to the Author” section, enter your conflict of interest statement in the “Confidential to Editor” section, and submit your "Accept" recommendation.

Reviewer #1: (No Response)

Reviewer #2: All comments have been addressed

**********

2. Is the manuscript technically sound, and do the data support the conclusions?

The manuscript must describe a technically sound piece of scientific research with data that supports the conclusions. Experiments must have been conducted rigorously, with appropriate controls, replication, and sample sizes. The conclusions must be drawn appropriately based on the data presented.

Reviewer #1: No

Reviewer #2: Yes

**********

3. Has the statistical analysis been performed appropriately and rigorously?

Reviewer #1: No

Reviewer #2: Yes

**********

4. Have the authors made all data underlying the findings in their manuscript fully available?

The PLOS Data policy requires authors to make all data underlying the findings described in their manuscript fully available without restriction, with rare exception (please refer to the Data Availability Statement in the manuscript PDF file). The data should be provided as part of the manuscript or its supporting information, or deposited to a public repository. For example, in addition to summary statistics, the data points behind means, medians and variance measures should be available. If there are restrictions on publicly sharing data—e.g. participant privacy or use of data from a third party—those must be specified.

Reviewer #1: No

Reviewer #2: Yes

**********

5. Is the manuscript presented in an intelligible fashion and written in standard English?

PLOS ONE does not copyedit accepted manuscripts, so the language in submitted articles must be clear, correct, and unambiguous. Any typographical or grammatical errors should be corrected at revision, so please note any specific errors here.

Reviewer #1: Yes

Reviewer #2: Yes

**********

6. Review Comments to the Author

Please use the space provided to explain your answers to the questions above. You may also include additional comments for the author, including concerns about dual publication, research ethics, or publication ethics. (Please upload your review as an attachment if it exceeds 20,000 characters)

Reviewer #1: 1- The graphical abstract still displays R²=0.99 for TN and R²=0.98 for COD, while the revised manuscript text now reports R²=0.83 and R²=0.76 respectively. The graphical abstract must be regenerated from the revised computational outputs.

2- The authors claim in their rebuttal to have implemented “nested cross-validation” and “study-level grouped cross-validation,” yet Section 2.3 of the revised manuscript still describes a standard 70/30 train-validation split with 5-fold CV and five repeats. There is no description of inner/outer loops, no mention of study-level grouping, and no reference to GroupKFold or equivalent implementation. The methods text directly contradicts the rebuttal claims. The authors should consult literature for proper implementation and reporting of grouped CV strategies for clustered environmental data.

3- The NLR formula correction and the shift from the rate constant k to removal efficiency as the prediction target are presented in the rebuttal as typographical fixes that did not affect the actual computational pipeline. This is a substantial claim; the authors are asserting that the original equations were wrong in the text but correct in their code, and that downstream results remain valid. Without providing verifiable evidence (e.g., code commits, audit logs, or at minimum the corrected code in the GitHub repository), this claim cannot be independently assessed. The authors should ensure the public repository reflects the described pipeline in its entirety.

4- The citation base for the ML methodology is narrow and somewhat dated. The authors should engage with more recent literature on ML interpretability in water treatment contexts, automated ML with SHAP-based interpretation for pollutant removal, and ML combined with meta-heuristic optimization for wastewater systems. Additionally, the meta-analysis methodology would benefit from citing established references on effect size computation and Hedges’ g correction beyond the general introductory references currently provided. The SHAP interpretation sections should also draw on the broader interpretable ML literature to properly frame the distinction between model explanation and causal inference. References the authors may wish to consult: Introduction to Meta-Analysis. Wiley, (2009).

Roberts, D.R. et al. Cross-validation strategies for data with temporal, spatial, hierarchical, or phylogenetic structure. Ecography, 40(8), 913–929, (2017); Interpretable Machine Learning: A Guide for Making Black Box Models Explainable. 2nd ed., (2022); A review on applications of biochar in constructed wetlands. Journal of Cleaner Production, 394, 136257, (2023); Machine Learning for Electrochemical Advanced Oxidation in Water Treatment: Descriptors, Interpretability, and Control. ACS ES&T Water, (2026); Automated machine learning and SHAP-based interpretation of PFOA removal via electrochemical oxidation. Desalination and Water Treatment, 101598, (2025).

5- The response to Reviewer #2’s Comment 8 regarding database and keyword bias is a complete non-answer. The reviewer asked specifically about the limitation of restricting the literature search to Web of Science with a narrow keyword set. The authors responded with a generic statement about improving clarity and streamlining content, entirely dodging the substantive concern. While the revised Limitations section does now briefly mention the WoS restriction, the evasiveness of the rebuttal response is concerning and suggests the authors may not have fully engaged with the underlying methodological critique.

Reviewer #2: (No Response)

**********

7. PLOS authors have the option to publish the peer review history of their article (what does this mean?). If published, this will include your full peer review and any attached files.

If you choose “no”, your identity will remain anonymous but your review may still be made public.

**Do you want your identity to be public for this peer review?**  For information about this choice, including consent withdrawal, please see our Privacy Policy.

Reviewer #1: No

Reviewer #2: No

**********

[NOTE: If reviewer comments were submitted as an attachment file, they will be attached to this email and accessible via the submission site. Please log into your account, locate the manuscript record, and check for the action link "View Attachments". If this link does not appear, there are no attachment files.]

To ensure your figures meet our technical requirements, please review our figure guidelines: https://journals.plos.org/plosone/s/figures

You may also use PLOS’s free figure tool, NAAS, to help you prepare publication quality figures: https://journals.plos.org/plosone/s/figures#loc-tools-for-figure-preparation.

NAAS will assess whether your figures meet our technical requirements by comparing each figure against our figure specifications.

# Author comment: Author response to Decision Letter 2

**DOI:** 10.1371/journal.pone.0353064.r004

30 May 2026

May 7th, 2026

Dear Editor and Reviewers:

Enclosed please find the revised manuscript (PONE-D-26-02227), “Pollution Removal Efficiency Enhancement by Agricultural Biomass Additions in Constructed Wetlands: A Framework Integrating Meta-Analysis with Explainable Machine Learning” by Wenjie Li, Jian Wang, Xinya Liu, Yulin Zhuang, Hongda Fang, Jinliang Huang. We appreciate the time and efforts you and the reviewers have dedicated to providing valuable feedback on our manuscript. We have ensured that each comment has been carefully addressed and the revised manuscript is now clearer and stronger. We are now submitting it for further consideration for publication in your esteemed journal. Please note that the line number in each response corresponds to the line number in the revised manuscript with marked changes.

Reviewer #1:

General comments:

1. The graphical abstract still displays R2=0.99 for TN and R2=0.98 for COD, while the revised manuscript text now reports R2=0.83 and R²=0.76 respectively. The graphical abstract must be regenerated from the revised computational outputs.

Response: We sincerely apologize for this oversight. You are absolutely correct. While we thoroughly updated the manuscript text and the main figures to reflect the new cross-validated results, we neglected to synchronize the Graphical Abstract. We have now regenerated the Graphical Abstract using the revised computational outputs. The updated image accurately displays the corrected performance metrics (R2=0.83 for the XGBoost model simulating TN removal, and R2=0.76 for the Random Forest model simulating COD removal), fully aligning with the main text. We deeply appreciate your meticulous attention to detail, which has helped us ensure complete consistency throughout all components of our submission.

2. The authors claim in their rebuttal to have implemented “nested cross-validation” and “study-level grouped cross-validation,” yet Section 2.3 of the revised manuscript still describes a standard 70/30 train-validation split with 5-fold CV and five repeats. There is no description of inner/outer loops, no mention of study-level grouping, and no reference to GroupKFold or equivalent implementation. The methods text directly contradicts the rebuttal claims. The authors should consult literature for proper implementation and reporting of grouped CV strategies for clustered environmental data.

Response: We sincerely apologize for this glaring discrepancy and deeply appreciate your meticulous review. You are absolutely right to call this out. While we had indeed completely overhauled our code to implement the nested and study-level grouped cross-validation prior to our previous submission, we unfortunately failed to update the corresponding methodology text in Section 2.3. It was a pure drafting oversight on our part, which regrettably left the outdated "70/30 split" text in the manuscript, directly contradicting our rebuttal claims. We have now completely rewritten Section 2.3 (Implementation of ML models) to accurately and transparently describe the computational pipeline that was actually executed. We have checked the manuscript to ensure it is now perfectly aligned with our actual methodology and the provided code repository (https://github.com/leeeee-max/Plos-One.git). Thank you for holding us to the highest standards of reporting accuracy; your sharp attention to detail has been invaluable in finalizing this manus

3. The NLR formula correction and the shift from the rate constant k to removal efficiency as the prediction target are presented in the rebuttal as typographical fixes that did not affect the actual computational pipeline. This is a substantial claim; the authors are asserting that the original equations were wrong in the text but correct in their code, and that downstream results remain valid. Without providing verifiable evidence (e.g., code commits, audit logs, or at minimum the corrected code in the GitHub repository), this claim cannot be independently assessed. The authors should ensure the public repository reflects the described pipeline in its entirety.

Response: We sincerely thank the reviewer for this sharp, highly justified, and necessary critique. You are absolutely correct to question our previous characterization of these critical changes as mere "typographical fixes." We deeply apologize for our poor choice of words in the previous rebuttal, which inadvertently downplayed the reality and extent of the revisions we performed.

To be completely transparent regarding our workflow and correct the record:

During our original manuscript submission, we did not provide the underlying code. At that time, the original manuscript indeed contained the flawed NLR formula and erroneously targeted the first-order rate constant k.

However, during the first round of revisions, directly prompted by the rigorous and constructive feedback from the reviewers. we conducted a comprehensive overhaul of our actual computational pipeline. We did not simply fix typos; we explicitly rewrote the programming scripts to correct the NLR formula (removing the redundant HRT term entirely) and fundamentally shifted the machine learning prediction target exclusively to absolute removal percentages. Therefore, the updated model outputs reported in our revised manuscript, specifically the R2=0.76 for COD and R2=0.82 for TN are the direct computational results of this newly overhauled and executed code, not the original pipeline. We apologize again for the confusion caused by our previous rebuttal wording.

4. The citation base for the ML methodology is narrow and somewhat dated. The authors should engage with more recent literature on ML interpretability in water treatment contexts, automated ML with SHAP-based interpretation for pollutant removal, and ML combined with meta-heuristic optimization for wastewater systems. Additionally, the meta-analysis methodology would benefit from citing established references on effect size computation and Hedges’ g correction beyond the general introductory references currently provided. The SHAP interpretation sections should also draw on the broader interpretable ML literature to properly frame the distinction between model explanation and causal inference. References the authors may wish to consult: Introduction to Meta-Analysis. Wiley, (2009).

Roberts, D.R. et al. Cross-validation strategies for data with temporal, spatial, hierarchical, or phylogenetic structure. Ecography, 40(8), 913-929, (2017); Interpretable Machine Learning: A Guide for Making Black Box Models Explainable. 2nd ed., (2022); A review on applications of biochar in constructed wetlands. Journal of Cleaner Production, 394, 136257, (2023); Machine Learning for Electrochemical Advanced Oxidation in Water Treatment: Descriptors, Interpretability, and Control. ACS ES&T Water, (2026); Automated machine learning and SHAP-based interpretation of PFOA removal via electrochemical oxidation. Desalination and Water Treatment, 101598, (2025).

Response: Thank you for your suggestion. We are deeply grateful to the reviewer for this sharp and highly critical observation. We fully agree that the initial R2 values of 0.98 were a clear red flag. Upon rigorous re-evaluation, we identified that this artificially inflated performance was caused by a combination of mathematical coupling in the feature space and data leakage stemming from standard random data splitting across clustered literature datasets. To address this fundamental methodological flaw, we have completely overhauled our machine learning pipeline and implemented a rigorous, leakage-free validation strategy in the revised manuscript. We have abandoned the previous ambiguous train-validation split. Instead, we established a strict nested cross-validation framework. Hyperparameter tuning is now strictly confined to the inner cross-validation loop. The optimized model is then evaluated exclusively on the completely untouched outer loop hold-out sets. This guarantees an absolute separation between model tuning and out-of-sample validation. To prevent data leakage where observations from the same primary study appear in both the training and testing sets, we replaced simple random splitting with Study-Level Grouped Cross-Validation. The models are now trained on a subset of studies and tested on entirely unseen studies, ensuring that the validation metrics reflect true external generalization. We have updated the Results section (Section 3.2), the Abstract, and the relevant Figures to reflect these new metrics. Furthermore, we have completely removed exaggerated claims such as "optimal" or "outstanding" from the text. We sincerely thank the reviewer for guiding us to significantly improve the rigor of our computational framework.

We sincerely appreciate this highly constructive feedback and thank you for providing such an excellent and highly relevant list of recommended literature. We fully agree that our original citation base was too narrow and did not adequately capture the most recent methodological advancements or the foundational statistical texts required to rigorously frame our study. Following your excellent suggestions, we have comprehensively updated our bibliography and enriched the corresponding discussions throughout the revised manuscript. Specifically, we have integrated your recommended references (alongside other recent studies) into the following sections:

Meta-Analysis Methodology (Section 2.2): We have cited the foundational text (Introduction to Meta-Analysis, Wiley, 2009) to provide a rigorous and established theoretical basis for our effect size computation and the specific application of the Hedges' g correction for small sample sizes.

Cross-Validation Strategy (Section 2.3): To properly ground our newly implemented study-level grouped cross-validation strategy, we have explicitly cited Roberts et al. (2017). This accurately justifies our approach to handling the hierarchical and clustered structure of the environmental data extracted from the primary studies.

Advanced ML in Water Treatment (Sections 1 & 4): We have engaged with the recent 2025 and 2026 literature you recommended (concerning automated ML, SHAP interpretation, and advanced oxidation in water treatment). Incorporating these state-of-the-art studies has significantly broadened our framing and demonstrated the wider applicability of explainable ML frameworks in wastewater systems.

SHAP Interpretation vs. Causal Inference (Sections 2.4 & 4): Drawing upon the Interpretable Machine Learning (2022) text, we have fundamentally refined our language regarding the SHAP analysis. We now use this literature to explicitly frame our SHAP results strictly as "model explanation" and "model-inferred feature importance," clearly distinguishing it from true physical or biogeochemical causal inference.

We are extremely grateful for these reading recommendations. They have significantly elevated the academic rigor, contextual depth, and current relevance of our methodological framework.

5. The response to Reviewer #2’s Comment 8 regarding database and keyword bias is a complete non-answer. The reviewer asked specifically about the limitation of restricting the literature search to Web of Science with a narrow keyword set. The authors responded with a generic statement about improving clarity and streamlining content, entirely dodging the substantive concern. While the revised Limitations section does now briefly mention the WoS restriction, the evasiveness of the rebuttal response is concerning and suggests the authors may not have fully engaged with the underlying methodological critique.

Response: We sincerely thank the reviewer for pointing out this glaring omission. You are absolutely correct that our previous response to Reviewer #2’s Comment 8 was a complete non-answer. We offer our deepest apologies for this. It was a highly regrettable clerical and drafting error during the compilation of the previous rebuttal: we accidentally pasted a generic formatting response (intended for a language/structure comment) into that section, completely overwriting our substantive explanation. We fully understand why this appeared evasive, and we assure you it was not our intention to dodge this critical methodological critique.

Our initial decision to restrict the search exclusively to the Web of Science (WoS) Core Collection was driven by the strict data quality requirements of our explainable machine learning framework. We required studies that reported highly detailed and standardized operational matrices (e.g., precise wetland volumes, HRT, and biomass physicochemical properties). However, we fully agree that this single-database approach introduces an inevitable database bias. It likely excluded highly relevant and high-quality empirical studies indexed in Scopus, Engineering Village, or regional databases (such as CNKI), potentially skewing the findings toward specific geographic regions or dominant research groups. To demonstrate our full engagement with this crucial methodological limitation, we have significantly expanded the "Limitations and future perspectives" section (Section 4.3) in the revised manuscript. The revised text now deeply reflects on how these database and keyword biases may impact the generalizability of our findings. We are extremely grateful for your rigorous oversight in ensuring this limitation is properly documented.

Attachment Submitted filename: Response to Reviews_2.docx

# Peer review: Decision Letter 2

**DOI:** 10.1371/journal.pone.0353064.r005

**Authors:** Sovik Das, Sovik Das, Sovik Das

18 Jun 2026

Pollution removal efficiency enhancement by agricultural biomass additions in constructed wetlands: A framework integrating meta-analysis with explainable machine learning

PONE-D-26-02227R2

Dear Dr. Huang,

We’re pleased to inform you that your manuscript has been judged scientifically suitable for publication and will be formally accepted for publication once it meets all outstanding technical requirements.

Within one week, you’ll receive an e-mail detailing the required amendments. When these have been addressed, you’ll receive a formal acceptance letter and your manuscript will be scheduled for publication.

An invoice will be generated when your article is formally accepted. Please note, if your institution has a publishing partnership with PLOS and your article meets the relevant criteria, all or part of your publication costs will be covered. Please make sure your user information is up-to-date by logging into Editorial Manager at Editorial Manager® and clicking the ‘Update My Information' link at the top of the page. For questions related to billing, please contact billing support.

If your institution or institutions have a press office, please notify them about your upcoming paper to help maximize its impact. If they’ll be preparing press materials, please inform our press team as soon as possible -- no later than 48 hours after receiving the formal acceptance. Your manuscript will remain under strict press embargo until 2 pm Eastern Time on the date of publication. For more information, please contact onepress@plos.org.

Kind regards,

Sovik Das

Academic Editor

PLOS One

Additional Editor Comments (optional):

Reviewers' comments:

Reviewer's Responses to Questions

**Comments to the Author**

1. If the authors have adequately addressed your comments raised in a previous round of review and you feel that this manuscript is now acceptable for publication, you may indicate that here to bypass the “Comments to the Author” section, enter your conflict of interest statement in the “Confidential to Editor” section, and submit your "Accept" recommendation.

Reviewer #1: All comments have been addressed

**********

2. Is the manuscript technically sound, and do the data support the conclusions?

The manuscript must describe a technically sound piece of scientific research with data that supports the conclusions. Experiments must have been conducted rigorously, with appropriate controls, replication, and sample sizes. The conclusions must be drawn appropriately based on the data presented.

Reviewer #1: (No Response)

**********

3. Has the statistical analysis been performed appropriately and rigorously?

Reviewer #1: (No Response)

**********

4. Have the authors made all data underlying the findings in their manuscript fully available?

The PLOS Data policy requires authors to make all data underlying the findings described in their manuscript fully available without restriction, with rare exception (please refer to the Data Availability Statement in the manuscript PDF file). The data should be provided as part of the manuscript or its supporting information, or deposited to a public repository. For example, in addition to summary statistics, the data points behind means, medians and variance measures should be available. If there are restrictions on publicly sharing data—e.g. participant privacy or use of data from a third party—those must be specified.

Reviewer #1: (No Response)

**********

5. Is the manuscript presented in an intelligible fashion and written in standard English?

PLOS ONE does not copyedit accepted manuscripts, so the language in submitted articles must be clear, correct, and unambiguous. Any typographical or grammatical errors should be corrected at revision, so please note any specific errors here.

Reviewer #1: (No Response)

**********

6. Review Comments to the Author

Please use the space provided to explain your answers to the questions above. You may also include additional comments for the author, including concerns about dual publication, research ethics, or publication ethics. (Please upload your review as an attachment if it exceeds 20,000 characters)

Reviewer #1: (No Response)

**********

7. PLOS authors have the option to publish the peer review history of their article (what does this mean?). If published, this will include your full peer review and any attached files.

If you choose “no”, your identity will remain anonymous but your review may still be made public.

**Do you want your identity to be public for this peer review?**  For information about this choice, including consent withdrawal, please see our Privacy Policy.

Reviewer #1: No

**********

# Editor report: Acceptance letter

**DOI:** 10.1371/journal.pone.0353064.r006

**Authors:** Sovik Das, Sovik Das, Sovik Das

PONE-D-26-02227R2

PLOS One

Dear Dr. Huang,

I'm pleased to inform you that your manuscript has been deemed suitable for publication in PLOS One. Congratulations! Your manuscript is now being handed over to our production team.

At this stage, our production department will prepare your paper for publication. This includes ensuring the following:

* All references, tables, and figures are properly cited

* All relevant supporting information is included in the manuscript submission,

* There are no issues that prevent the paper from being properly typeset

You will receive further instructions from the production team, including instructions on how to review your proof when it is ready. Please keep in mind that we are working through a large volume of accepted articles, so please give us a few days to review your paper and let you know the next and final steps.

Lastly, if your institution or institutions have a press office, please let them know about your upcoming paper now to help maximize its impact. If they'll be preparing press materials, please inform our press team within the next 48 hours. Your manuscript will remain under strict press embargo until 2 pm Eastern Time on the date of publication. For more information, please contact onepress@plos.org.

You will receive an invoice from PLOS for your publication fee after your manuscript has reached the completed accept phase. If you receive an email requesting payment before acceptance or for any other service, this may be a phishing scheme. Learn how to identify phishing emails and protect your accounts at https://explore.plos.org/phishing.

If we can help with anything else, please email us at customercare@plos.org.

Thank you for submitting your work to PLOS ONE and supporting open access.

Kind regards,

PLOS ONE Editorial Office Staff

on behalf of

Prof Sovik Das

Academic Editor

PLOS One
